# Specificity and dynamics of H_2_O_2_ detoxification by the cytosolic redox regulatory network as revealed by *in vitro* reconstitution

**DOI:** 10.1016/j.redox.2024.103141

**Published:** 2024-04-03

**Authors:** Lara Vogelsang, Jürgen Eirich, Iris Finkemeier, Karl-Josef Dietz

**Affiliations:** a Biochemistry and Physiology of Plants, Faculty of Biology, Bielefeld University, 33615, Bielefeld, Germany; b CeBiTec, Bielefeld University, 33615, Bielefeld, Germany; c Plant Physiology, Institute of Plant Biology and Biotechnology, University of Münster, 48149, Münster, Germany

**Keywords:** Glutathione peroxidase, Hydrogen peroxide, Peroxiredoxin, Reactive oxygen species, Thioredoxin

## Abstract

The thiol redox state is a decisive functional characteristic of proteins in cell biology. Plasmatic cell compartments maintain a thiol-based redox regulatory network linked to the glutathione/glutathione disulfide couple (GSH/GSSG) and the NAD(P)H system. The basic network constituents are known and *in vivo* cell imaging with gene-encoded probes have revealed insight into the dynamics of the [GSH]^2^/[GSSG] redox potential, cellular H_2_O_2_ and NAD(P)H+H^+^ amounts in dependence on metabolic and environmental cues. Less understood is the contribution and interaction of the network components, also because of compensatory reactions in genetic approaches. Reconstituting the cytosolic network of *Arabidopsis thaliana in vitro* from fifteen recombinant proteins at *in vivo* concentrations, namely glutathione peroxidase-like (GPXL), peroxiredoxins (PRX), glutaredoxins (GRX), thioredoxins, NADPH-dependent thioredoxin reductase A and glutathione reductase and applying Grx1-roGFP2 or roGFP2-Orp1 as dynamic sensors, allowed for monitoring the response to a single H_2_O_2_ pulse. The major change in thiol oxidation as quantified by mass spectrometry-based proteomics occurred in relevant peptides of GPXL, and to a lesser extent of PRX, while other Cys-containing peptides only showed small changes in their redox state and protection. Titration of ascorbate peroxidase (APX) into the system together with dehydroascorbate reductase lowered the oxidation of the fluorescent sensors in the network but was unable to suppress it. The results demonstrate the power of the network to detoxify H_2_O_2_, the partially independent branches of electron flow with significance for specific cell signaling and the importance of APX to modulate the signaling without suppressing it and shifting the burden to glutathione oxidation.

## 1 Introduction

The activities of most, if not all, cellular processes rely on the proper redox milieu within involved cell compartments and the redox state of the associated proteins. Examples are gene expression that is affected by redox-controlled transcription factors, protein synthesis modulated by redox-dependent translation factors, metabolism governed by redox-sensitive enzymes, and cell signaling regulated by redox-responsive signal transduction elements [[1], [2], [3], [4]].

The redox factor affecting protein function with high prevalence is related to cysteinyl thiols and their redox derivatives. Protein thiols in plasmatic compartments, i.e., the cytosol, nucleoplasma, matrix and stroma, commonly are reduced, and adopt more oxidized thiol states such as intra- or intermolecular disulfide, sulfenyl, sulfinyl, sulfonyl and S-nitrosylated forms in response to certain developmental or environmental cues [5]. The oxidation is linked to reactive oxygen species (ROS) or other reactive molecular species that function as final electron acceptors, and their generator systems [6]. Protein and non-protein thiols are part of a thiol redox regulatory network that controls the various cellular processes described above [[7], [8], [9], [10]].

The redox network consists of input elements, transmitters, sensors and target proteins [7]. However, the analysis of the state of the redox network *in vivo* and the assignment of specific functions to its elements are hampered by yet unsolved drawbacks in assessing redundancy, specificity, and alternative compensatory pathways. This work focuses on the flowering plant *Arabidopsis thaliana* and uses the protein nomenclature of plants. Fundamental questions concern the role of and the coupling between the thiol peroxidases peroxiredoxin (PRX) and glutathione peroxidase-like (GPXL), the thioredoxin (TRX) and glutaredoxin (GRX) isoforms and the redox-regulated protein targets. In the cytosol, the type II PRXIIB/C/D are most efficiently coupled to GRX, GSH, glutathione reductase (GR) and NADPH, while GPXL2/8 retrieve electrons from the TRX and NADPH-dependent TRX reductase A/B (NTRA/B) and NADPH [11].

Genetic approaches using loss of function mutants have revealed that elements and pathways within the network are redundant. Deletion mutants lacking the cytosolic and mitochondrial NTRA and NTRB were viable displaying only a few phenotypic alterations like wrinkled seeds [12], while TRXs remained partly reduced. However, after additional blocking of glutathione synthesis by treatment with buthionine sulfoximine, TRXh3 was fully oxidized. This kind of genetic approach apparently has limitations that urge researchers to develop additional approaches.

Highly sensitive proteomics nowadays allows for the detection of posttranslational modifications (PTM) *ex vivo*, i.e., in extracts prepared from cells and tissues [13,14]. However, specificity, sensitivity, and coverage of functional thiols within polypeptides of interest remain technologically challenging. To assess dithiol changes between treatments or mutants in mass spectrometry-based proteomic approaches in a quantitative manner, it is mandatory to use isotopic labels on cysteines, such as Iodoacetyl Tandem Mass Tag™ (iodoTMT™) or light and heavy compounds for example [[15], [16], [17]]. Biotin switch-based methods where in case of S-nitrosylation ascorbate releases the nitric oxide, or in case of S-glutathionylation a monocysteinic glutaredoxins (GRX) releases the glutathione, and the freed thiol is labeled with thiol labeling probes, have revealed sets of proteins that, e.g., are glutathionylated *in vivo*, e.g., in *Chlamydomonas reinhardtii* [18]. However, the background noise and the variability due to the multistep detection procedure are drawbacks in reliably detecting glutathionylated proteins under non-stressed conditions [19,20]. In parallel, the portfolio of methods available for redox proteomics, e.g., for identifying sulfenylated proteins from live samples, has increased over the last years and has allowed for assessing the sulfenylome [21]. Sulfenylated proteins are the first products if H_2_O_2_ reacts with thiols. Such approaches trap and accumulate possible primary targets of H_2_O_2_, but do not easily allow for quantification or rate determination.

The development and availability of gene-encoded fluorescent sensors enables researchers monitoring central parameters of the redox regulatory network *in vivo*. Commonly used tools for H_2_O_2_ quantification are based on fusion of roGFP2 with the yeast thiol peroxidase ORP1 [22], and the sensor for glutathione redox state was constructed as fusion of roGFP2 with GRX1 [23]. As these gene-encoded sensors can be targeted to different cell compartments, they allow for determining changes in H_2_O_2_ levels and the redox potential of the glutathione redox couple [GSH]^2^/[GSSG] with spatial and temporal resolution. In case of the roGFP2-ORP1 sensor, it should be kept in mind that the readout of fluorescence is a qualitative measure since the rate of regeneration of reduced roGFP2 as here in plants depends on not well-defined redox transmitters and uncharacterized rates of electron transfer.

Given the significant methodological progress in recent years, and the persistent challenges in fully elucidating the interconnections within the redox regulatory network, this work sought to develop an innovative experimental approach by reconstituting a partial network of the cytosol *in vitro*. By combining the reconstituted system with H_2_O_2_- and GSH/GSSG-redox sensors and quantitative redox proteomics, the study was expected to unravel the dynamics and connectivity between the network components in a realistic scenario. By omitting elements or introducing ascorbate peroxidase, we aimed for monitoring shifting burden in H_2_O_2_ detoxification and protection of the thiol system in a unique manner. Thereby, the results provide fundamental insight into the dynamics of the network and is the basis for interpreting results from *in vivo* studies that fail to dissect the network interference.

## 2 Materials and methods

### 2.1 Molecular cloning of coding sequences for heterologous expression

For recombinant protein expression, the coding sequences of *A. thaliana* DHAR1 (AT1G19570) and APX2 (AT3G09640) were amplified from leaf cDNA using specific forward and reverse primers with restriction sites (Suppl. Table S2) and cloned into pET28a. The expression vectors for PRXIID (AT1G60740), GPXL2 (AT2G31570), TRXh1 (AT3G51030), TRXh2 (AT5G39950), TDX (AT3G17880) and GRXC1 (AT5G63030) were cloned into pET28a as described in Vogelsang & Dietz [11]. PRXIIB (AT1G65980), GPXL8 (AT1G63460), TRXh3 (AT5G42980), TRXh5 (AT1G45145), GRXC2 (AT5G40370), NTRA (AT2G17420, without the sequence coding for the transit peptide), GR (AT3G24170) and PGK3 (AT1G79550) were cloned into pET28a as described in Knieper et al. [24]. MDH1 (AT1G04410) and GAPC2 (AT1G13440) in pET16b were kindly provided by the working group of Renate Scheibe [25,26] and Grx1-roGFP2 in pQE60 and roGFP2-Orp1 in pETG10A by Tobias Dick and Andreas Meyer [27,28]. Both sensors were also cloned into CaMV35S-eYFP [29] for transient expression in protoplasts. To this end, roGFP2-Orp1 and Grx1-roGFP2 were amplified from their expression vectors using specific forward and reverse primers with restriction sites (Suppl. Table S2) and eYFP was replaced by the sensor using NcoI and NotI restriction sites. The CaMV35S-promoter was replaced by the Arabidopsis ubiquitin 10 promoter using HindIII and BamHI restriction sites. For FRET-measurements, PRXIIB (AT1G65980), GPXL2 (AT2G31570), GAPC2 (AT1G13440) and MDH1 (AT1G04410) were cloned in both CaMV35S-mTurquoise2-NosT and CaMV35S-eYFP-NosT [29,30] using BamHI and AgeI after amplifying them from their expression constructs (see Suppl. Table S2 for primers). All sequences were verified by DNA sequencing.

### 2.2 Protein expression and purification

For recombinant protein expression, either *Escherichia coli* BL21 (DE3) pLysS, NiCo21(DE3) or Rosetta-gami 2(DE3) pLysS cells were transformed with plasmid DNA (Suppl. Table S3). After inducing protein expression with 400 μM isopropyl-β-d-thiogalactopyranoside (or 1 mM in case of Grx1-roGFP2) in the exponential phase, expression was carried out for 4 h at 37 °C and 170 rpm. Bacteria were harvested by centrifugation (11,000*×g*, 10 min, 4 °C) and resuspended in lysis buffer (50 mM Tris-HCl, pH 8, 300 mM NaCl, 5 mM imidazole, 10 mM β-mercaptoethanol (βME), 1 mM phenylmethylsulfonyl fluoride (PMSF)). Cells were lysed by adding 200 μg/mL lysozyme for 30 min at 20 °C, followed by sonication. Soluble and insoluble fractions were separated by centrifugation at 20,000*×g* and 4 °C for 60 min. The lysate was incubated with Roti®Garose-His/Ni NTA-beads (Carl Roth, Karlsruhe, Germany) previously equilibrated with lysis buffer, under slow shaking at 4 °C for 60 min. After washing with 200 mL washing buffer I (50 mM Tris-HCl, pH 8, 300 mM NaCl, 10 mM imidazole) and 50 mL washing buffer II (50 mM Tris-HCl, pH 8, 300 mM NaCl, 20 mM imidazole), the His_6_-tagged protein was eluted using the elution buffer (50 mM Tris-HCl, pH 8, 300 mM NaCl, 250 mM imidazole, 10 mM βME, 1 mM PMSF). Eluted protein-containing fractions were pooled, concentrated, and dialyzed overnight at 4 °C against 40 mM K-Pi, pH 7.2 (dialysis tubing Membra-Cel ™, cellulose, flat width 25 mm, MWCO 14,000, Carl Roth) with repeated changes of dialysis buffer. Final purity was checked by separation in 12% (w/v) SDS-PAGE. Western blots were performed with primary antibodies directed against the His_6_-tag (Santa Cruz Biotechnology, sc-81045) and secondary antibodies conjugated with horseradish peroxidase (Sigma-Aldrich, A9044). Prior to use, proteins were centrifuged at 20,000×*g* for 5 min to remove protein aggregates. Protein concentration was determined photometrically using the protein-specific molar extinction coefficient at 280 nm calculated with Expasy-tool ProtParam [31].

### 2.3 Monitoring [GSH^2^/GSSG] redox potential and H_2_O_2_ level

3.6-fold concentrated proteins (1.25 μM GPXL2, 0.54 μM GPXL8, 0.57 μM NTRA, 0.54 μM GR, 0.54 μM TRXh1, 0.54 μM TRXh2, 5.68 μM TRXh3, 2.54 μM TRXh5, 0.54 μM GRXC1, 3.64 μM GRXC2, 3.39 μM PRXIIB, 0.54 μM PRXIID, 0.54 μM TDX, 26.50 μM GAPC2, 9.14 μM MDH1 and 0.36 μM roGFP2) were mixed in 40 mM KP_i_, pH 7.2, then kept on ice. Two identical mixtures were prepared, one containing roGFP2-Orp1 and the other one Grx1-roGFP2. One hour before the measurement, samples were diluted to 1-fold and supplemented with 500 μM GSH and 200 μM NADPH in a total volume of 195 μl. After 60 min at 21 °C and 300 rpm for equilibration, the samples were transferred to a well of a 96-well slide (μ-Plate 96 Well Black, uncoated (89621), ibidi) for analysis by confocal laser scanning microscopy. After recording the basal emission for 4 min, 5 μl of oxidant, namely H_2_O_2_, tBOOH, CuOOH or/and GSSG were added at concentrations as indicated and measurements were continued for 25 min. A Zeiss LSM780 confocal microscope with an objective of 10-fold magnification (Plan-Apochromat 10×/0.45) and main beam splitters MBS488 and MBS405 was used to detect roGFP2 in the range of 500–600 nm and excited sequentially by 405 nm- (15% laser power) and 488 nm-laser (5% laser power) lines. Images were obtained every 5.85 s, pixel dwell time was 5 μs. Pinhole was open for measurements of recombinant proteins and set to 1 AIRY unit for protoplasts. For data evaluation, identical regions of interest were defined, mean pixel values extracted and the ratio of emissions under 405 nm- and 488 nm-excitation was calculated.

The measurements were performed in absence of each one of the components PRX, GPXL, PRX and GPXL, TRX, GRX, TRX and GRX, NTRA, GR, NTRA and GR as well. Also, an additional set of measurements was performed with the reconstitution system expanded by APX2 and DHAR1. DHAR1 at 6.71 μM and APX2 at 18, 36, 90, 180, 360 nM or 3.6 μM concentration were added to the 3.6-fold concentrated protein mix. For equilibration, the mix was again diluted to 1-fold (1.88 μM DHAR1 and 5, 10, 25, 50, 100, 1000 nM APX2, other proteins as described above), but supplemented with 1 mM ASC, 1 mM GSH and 1 mM NADPH.

### 2.4 Measurement of NADPH concentration

NADPH oxidation was monitored in the reconstituted system after the addition of 100 μM H_2_O_2_ by spectrophotometry at 340 nm (Cary 3500 UV–Vis, Agilent, Santa Clara, CA, USA). The reconstitution mix contained 0.35 μM GPXL2, 0.15 μM GPXL8, 0.16 μM NTRA, 0.15 μM GR, 0.15 μM TRXh1, 0.15 μM TRXh2, 1.59 μM TRXh3, 0.71 μM TRXh5, 0.15 μM GRXC1, 1.02 μM GRXC2, 0.95 μM PRXIIB, 0.15 μM PRXIID, 0.15 μM TDX, 7.42 μM GAPC2 and 2.56 μM MDH1, supplemented with 500 μM GSH and 200 μM NADPH in 40 mM KP_i_, pH 7.2. Sample equilibration occurred at 21 °C and 500 rpm for 60 min and storage on ice. After recording the basal absorbance of 95 μl master mix at 340 nm for 4 min, addition of 5 μl 2 mM H_2_O_2_ initiated the reaction, i.e., 100 μM H_2_O_2_ final concentration or a pulse of 10 nmol H_2_O_2_. The recording proceeded for additional 25 min either in the complete set or in the absence of single components as indicated. NADPH oxidation was calculated based on *ε* = 6220 M^−1^cm^−1^.

### 2.5 Sample preparation for mass spectrometry

The 15 proteins of the reconstituted system were adjusted to a total concentration of 35 μM individually and reduced by 20 mM DTT at room temperature for 30 min. Subsequently, the samples were desalted by Zeba Spin desalting columns (Thermo Fisher Scientific) and the concentration checked again. 0.35 μM GPXL2, 0.15 μM GPXL8, 0.16 μM NTRA, 0.15 μM GR, 0.15 μM TRXh1, 0.15 μM TRXh2, 1.59 μM TRXh3, 0.71 μM TRXh5, 0.15 μM GRXC1, 1.02 μM GRXC2, 0.95 μM PRXIIB, 0.15 μM PRXIID, 0.15 μM TDX, 7.42 μM GAPC2 and 2.56 μM MDH1 were mixed in a total volume of 1.12 ml containing 560 μg total protein to be separated later on into 56 samples with 10 μg/20 μl each (four replicates, seven time points, two different alkylation procedures). An additional identical mixture was prepared without PRXIIB and PRXIID. Both samples were supplemented with 500 μM GSH, 200 μM NADPH and 140 μM NAD and were equilibrated for 1 h. Samples were split into 28 aliquots of 40 μl each with and without PRX.

To detect free and blocked thiols, two step-alkylation was performed with 55 mM N-ethylmaleimide (NEM) in the first and 55 mM deuterated-NEM (N-ethyl-d_5_-maleimide, D-6141, CDN Isotopes) in the second step allowing for distinguishing between reduced and oxidized thiols. The labeling order was reversed in half of the samples.

Alkylation was performed after different incubation times (0 s, 15s, 30 s, 60 s, 120 s, 7 min and 20 min) before or after addition of 100 μM H_2_O_2_, taking care of adjusting equal volumes. Thiols were alkylated by addition of 44 μl alkylation buffer (0.1 M Tris HCl pH 8, 1 mM CaCl_2_, 8 M urea, 110 mM NEM). All samples were incubated in the dark at 20 °C and 100 rpm for 30 min. NEM and H_2_O_2_ were removed by Zeba Spin desalting columns (Thermo Fisher Scientific). Samples were split into each two 40 μl aliquots and proteins precipitated by adding 4 vol of ice-cold acetone at −20 °C overnight. Samples were incubated at room temperature for 1 h and vortexed before centrifugation at 13.000*×g* and room temperature for 10 min. Pellets were washed twice with 200 μl 80 % acetone and once with 200 μl 100 % acetone, each time in between they were centrifuged for 5 min. Afterwards the pellets were dried in a vacuum concentrator. Half of the samples were stored at −20 °C until trypsin digestion, the other half was subjected to reduction and a second alkylation.

Samples were reduced by resuspending the pellets in 40 μl 0.1 M Tris HCl, pH 8, 1 mM CaCl_2_, 8 M urea, 1 mM TCEP and shaking for 30 min, followed by vortexing and sonification cycles of 30 s, 60 s and thrice 10 s. Alkylation was performed by addition of 8 μl 330 mM NEM (55 mM final) and an incubation in the dark at 20 °C and 100 rpm for 30 min. Proteins were precipitated by addition of 4 vol ice-cold acetone at −20 °C overnight. Pellets were washed and dried as described before.

All samples were resuspended in 50 mM ammonium bicarbonate by shaking for 45 min, vortexing and sonification for 30 s and thrice 10 s 20 μg Trypsin (Promega V5111) was resuspended in 400 μl Trypsin Resuspension Buffer and incubated at 30 °C for 15 min 2 μl of Trypsin (50 ng/μl) was added to each sample (1:100 trypsin: protein) and samples were incubated at 37 °C overnight. The reaction was stopped by addition of 0.5 % formic acid. Finally, all samples were dried in a vacuum concentrator.

### 2.6 LC-MS/MS and data acquisition analysis

Samples were analyzed using an EASY-nLC 1200 (Thermo Fisher) coupled to an Exploris 480 (E480) Mass Spectrometer ([32], Thermo Fisher). Peptides were separated and sprayed with 25 cm fused silica emitters (75 μm inner diameter, CoAnn Technologies) packed in-house with ReproSil-Pur 120 C_18_ AQ 1.9 μm (Dr. Maisch). 0.5 μg of peptides per sample were analyzed using a stepped gradient of 0 %–45 % solvent B (80 % ACN, 0.1 % FA) in 60 min at 250 μl/min, or 0 %–55 % solvent B in 100 min at 300 μl/min, followed by wash steps. Peptide survey mass spectra were acquired in the Orbitrap analyzer, with a resolution of 120,000 on the E480. A resolution of 15,000 for MS^2^ spectra was used on both instruments. The scanned mass range was 300–1750 *m/z*. The normalized collision energy was set to 25. Peptides with a charge of +1, > +6, or peptides with an unassigned charge state were excluded from fragmentation.

Processing of raw data was performed using the MaxQuant software version 2.1.3.0 [33]. MS/MS spectra were assigned to the *E. coli* Uniprot reference proteome supplemented with the sequences of the recombinantly expressed proteins. During the search, sequences of 248 common contaminant proteins as well as decoy sequences were automatically added. Trypsin specificity was required and a maximum of two missed cleavages was allowed. Heavy and light NEM on cysteine residues were used as MS^1^ labels. Oxidation of methionine, deamidation and protein N-terminal acetylation were set as variable modifications. A false discovery rate of 1% for peptide spectrum matches and proteins was applied. Match between runs, re-quantification and iBAQ were enabled. The MaxQuant table of peptides was imported into R. Intensities from heavy and light peptides were log_2_ transformed and used for analysis.

### 2.7 Measurement of MDH and GAPDH activity

Activity assays were carried out in three different ways: The first assay contained GAPC2/MDH1 and GSH and NADPH, the second GAPC2/MDH1 in the reconstituted system with GSH and NADPH and the third the reconstituted system with GSH and NADPH but without the target GAPC2/MDH1.

A 3.6-fold protein mix (1.25 μM GPXL2, 0.54 μM GPXL8, 0.57 μM NTRA, 0.54 μM GR, 0.54 μM TRXh1, 0.54 μM TRXh2, 5.68 μM TRXh3, 2.54 μM TRXh5, 0.54 μM GRXC1, 3.64 μM GRXC2, 3.39 μM PRXIIB, 0.54 μM PRXIID, 0.54 μM TDX, 26.50 μM GAPC2 and 9.14 μM MDH1) was supplemented with 140 μM NAD and 20 mM DTT in a total volume of 100 μl. The samples were incubated at 20 °C for 30 min and then desalted by Zeba Spin desalting columns (Thermo Fisher Scientific). The protein mix was diluted to 1-fold (0.35 μM GPXL2, 0.15 μM GPXL8, 0.16 μM NTRA, 0.15 μM GR, 0.15 μM TRXh1, 0.15 μM TRXh2, 1.59 μM TRXh3, 0.71 μM TRXh5, 0.15 μM GRXC1, 1.02 μM GRXC2, 0.95 μM PRXIIB, 0.15 μM PRXIID, 0.15 μM TDX, 7.42 μM GAPC2, 2.56 μM MDH1), supplemented with 500 μM GSH, 200 μM NADPH and 140 μM NAD and incubated at 20 °C and 500 rpm for 30 min, then kept on ice. H_2_O_2_ was added at final concentrations of 100 or 500 μM and the samples were incubated for 30 min at 20 °C.

For GAPDH-activity, 4 mM ATP, 8 mM 3-phosphoglycerate disodium salt (3-PGA), 0.26 mM NADH, 90 nM phosphoglycerate kinase (PGK), 8 mM MgSO_4_ and 1 mM EDTA in 100 mM Tris-HCl, pH 7.8, were preincubated at RT for 10 min, so that the GAPC2-substrate 1,3-bisphosphoglycerate was formed, and the mixture was degassed afterwards. The reaction mix (1 ml) was measured at RT in a quartz cuvette at 340 nm for 1 min before 2 μl of the sample were added to reach a final concentration of 15 nM GAPC2. The spectrophotometric measurement was continued for further 5 min (Cary 3500 UV–Vis, Agilent, Santa Clara, CA, USA).

For MDH-activity, samples were diluted 1:25 with 40 mM KP_i_, pH 7.2. In preparation for the measurement, 100 mM Tris, pH 7.5, 10 mM MgCl_2_, 0.1 mg/ml BSA, 0.2 mM NADH were degassed and 985 μl were transferred to a cuvette. 5 μl of diluted sample was added to reach a final concentration of 0.5 nM MDH1 and the basal absorbance was measured at 340 nm for 2 min. Reaction was started by addition of 10 μl 100 mM oxaloacetate (OAA, 1 mM final conc.) and absorbance was measured for additional 5 min.

To determine background oxidation of NADH, the assays were also performed in absence of proteins and NADPH and GSH. Measurements without GAPC2/MDH1 were subtracted from the measurements with GAPC2/MDH1. Enzyme activities were calculated using the molar extinction coefficient of NADH (*ε* = 6220 M^−1^cm^−1^).

### 2.8 Redox-dependent protein conformation

Redox-dependent protein conformation and aggregate formation was analyzed by non-reducing SDS-PAGE and silver nitrate staining as will be shown in Suppl. Fig. S6. 2-fold concentrated proteins (0.7 μM GPXL2, 0.3 μM GPXL8, 0.32 μM NTRA, 0.3 μM GR, 0.3 μM TRXh1, 0.3 μM TRXh2, 3.18 μM TRXh3, 1.42 μM TRXh5, 0.3 μM GRXC1, 2.04 μM GRXC2, 1.9 μM PRXIIB, 0.3 μM PRXIID, 0.3 μM TDX, 14.84 μM GAPC2 and 5.12 μM MDH1) were mixed with 20 mM DTT, incubated for 30 min at 21 °C and desalted by Zeba Spin desalting columns (Thermo Fisher Scientific). Protein mix was diluted to 1-fold, supplemented with 500 μM GSH, 200 μM NADPH and 140 μM NAD and incubated at 20 °C and 500 rpm for 30 min. Protein mixtures were subjected to 100 μM H_2_O_2_ and five samples were prepared, which were supplemented with 100 mM iodoacetamide (IAA) after 0, 0.25, 0.5, 2, 7 and 20 min, respectively. Samples were incubated in the dark at 20 °C and 200 rpm for 30 min. Lanes of the gel were loaded with either 5 μg or 25 μg total protein in non-reducing loading buffer (45 mM Tris, pH 6.8, 1% (w/v) SDS, 0,01% (w/v) bromophenol blue 10% (v/v) glycerol) without boiling. Proteins were separated by 12% (w/v) SDS-PAGE and subsequently visualized by silver staining.

### 2.9 FRET measurements of protein interactions

Protoplast isolation and transfection were performed as described before [30,34]. Leaves from 7-week-old *A. thaliana* plants, grown in short day, were used. FRET between mTurquoise2 and eYFP was measured by sensitized emission in transfected protoplasts [29,35]. The cloning of the constructs is described in the molecular cloning section above (1st paragraph in Material and Methods). Measurements were performed with untreated cells, reduced cells incubated with 1 mM DTT for 30 min and oxidized cells that were first reduced and then oxidized by adding 5 mM H_2_O_2_ for 30 min. For image acquisition, a Zeiss LSM5 with a Zeiss Plan-Neofluar 40×/0.75 water immersion objective has been used with the following settings: The main beam splitter was MBS458/514, mTurquoise2 and eYFP-emission were detected in the range of 470–510 nm and 530–600 nm, respectively, with pixel dwell times of 3–4 μs and a bit depth of 12 bit. The donor was excited using the 458 nm-line of an argon ion laser, the acceptor with the 514 nm-line of an argon ion laser in a sequential and line-wise manner.

### 2.10 Measurements of GSH-redox state and H_2_O_2_-abundance in protoplasts

The measurements applied the sensors Grx1-roGFP2 (GSH/GSSG redox state) and roGFP2-Orp1 (H_2_O_2_ abundance) under control of the *A. thaliana* ubiquitin 10 promoter. Technically, a Zeiss LSM780 confocal microscope with a water-immersion objective (Zeiss LCI Plan-Neofluar 63× /1.3 Imm. Korr. DIC M27) and main beam splitters MBS488 and MBS405 was applied. roGFP2 was detected in the range of 500–600 nm and excited sequentially by 405 nm- and 488 nm-laser lines. The pixel dwell time was around 3 μs and the bit depth was 12 bit. Maxima of the two line-profiles per cell and the ratios of emissions under 405 nm- and 488 nm-excitation were calculated.

### 2.11 Statistics

Significance of differences was either determined with the Student's *t*-test (p < 0.05) using Excel or ANOVA followed by Tukey post hoc test (p < 0–05) using IBM SPSS Statistics 20 or R.

## 3 Results

**Fig. 1:**
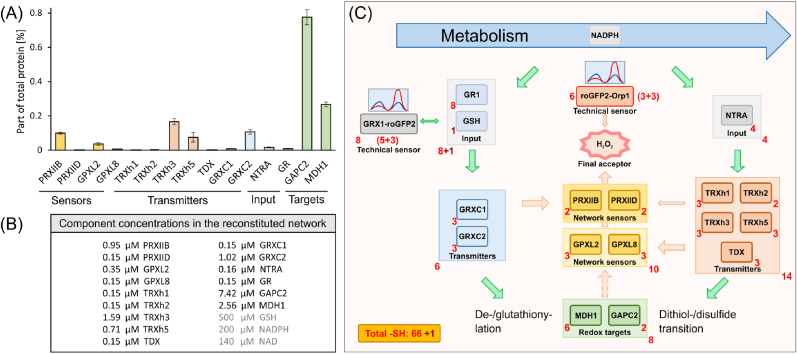
Choice of proteins for the reconstituted network. Thioredoxins such as TRXh7-9, which were inactive as electron donors for cytosolic thiol peroxidases [11], were excluded from the network. If tryptic digestion of protein isoforms resulted in identical peptides, the less active or less abundant isoform was omitted from the network, such as PRXIIC or TRXh4. (A) The relative abundance of redox sensors, transmitters, input elements as well as targets is shown. The data derives from proteomic data of *A. thaliana* leaf samples. To gain a general abundance, samples were taken from plants grown under control conditions and plants stressed by arsenic, hypoxia, and an arsenic-hypoxia stress combination. The data is provided as mean iBAQ-value relative to the sum of the iBAQ-values of all identified proteins, e.g., GAPC2 represented 0.78 % of total protein (for details and data repository see Suppl. Table S4). Mean ± SE, n = 4. (B) Final protein concentrations for the network reconstitution based on the data shown in (A). Proteins of low concentration were set to a minimum concentration of 0.15 μM so that the maximum abundance ratio of the proteins was 50 and a sample concentration of ≥10 μg/20 μl was ensured. Besides the protein concentrations, the concentrations of GSH, NADPH and NAD used for the network reconstitution are provided. (C) Reconstitution of the cytosolic redox-regulatory network. The schematics depicts all components present in the standard network. The red numbers denote the Cys thiols in each redox element. (For interpretation of the references to colour in this figure legend, the reader is referred to the Web version of this article.)

The cytosolic thiol redox-regulatory network of *Arabidopsis thaliana* cells was reconstituted based on the following data and assumptions: (i) Quantitative proteomics of rosettes of *A. thaliana* provided information on relative quantities of network elements as shown in Fig. 1A, a list of all used and mentioned proteins, their Arabidopsis genome identifiers (AGI), and their abbreviations can be found in Suppl. Table S1. (ii) Abundant polypeptides were added in accordance with this data, however, all polypeptides equal or below 0.15 μM were added at 0.15 μM concentration (Fig. 1B). (iii) Both fluorescent sensors were added alternatively at a concentration of 0.1 μM. Due to their similar excitation and emission characteristics, roGFP2-Orp1 and Grx1-roGFP2 could not be added simultaneously. (iv) In order to allow for distinguishing the elements by proteomics and avoid redundant peptides, we reduced the complexity to the 15 + 1 polypeptides as indicated. Fig. 1C depicts the network with its tentative wiring.

**Fig. 2:**
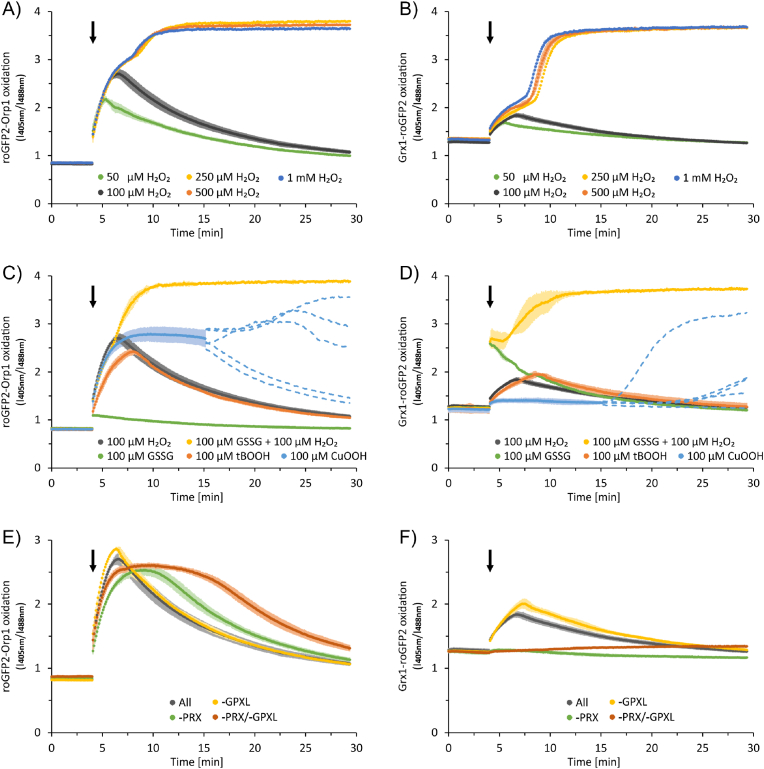
Changes in H_2_O_2_ concentration and E[GSH]^2^/[GSSG] in the reconstituted network including 500 μM GSH and 200 μM NADPH monitored by roGFP2 coupled to Orp1 or GRX1. Oxidation state was measured by laser scanning microscopy as ratio of the fluorescence emissions at the excitation wavelengths 405 nm and 488 nm. The emissions were recorded for 30 min. The first 4 min show baseline recordings, then oxidants were added as indicated (arrow). (A, B) Response of roGFP2-Orp1 and Grx1-roGFP2 to increasing H_2_O_2_-concentrations, respectively. (C, D) Comparison of the roGFP2-Orp1 and Grx1-roGFP2 oxidation state after addition of the different oxidants H_2_O_2_, GSSG, GSSG and H_2_O_2_, tBOOH and CuOOH, respectively. (E, F) Impact of the presence or absence of thiol peroxidases in the reconstitution network on the oxidation state of the sensors roGFP2-Orp1 and Grx1-roGFP2, respectively. The redox state was monitored in the absence of either GPXL or PRX or GPXL/PRX. Mean ± SD is shown, n = 2–10.

At the time indicated, H_2_O_2_ at various concentrations was added as a single injection (Fig. 2A and B). Both the roGFP2-Orp1 and the Grx1-roGFP2 sensors rapidly turned oxidized. The principal shapes of sensor response curves after introducing H_2_O_2_ resembled each other with a rise to a maximum followed by recovery over the subsequent 25 min. However, the amplitude differed greatly with much stronger oxidation of the roGFP2-Orp1 sensor for H_2_O_2_ than the Grx1-roGFP2 sensor sensing E[GSH]^2^/[GSSG]. The occurrence of the maximal sensor oxidation and the half time of recovery matched between both sensors, with the roGFP2-Orp1 being slightly ahead. The difference in amplitude and kinetics agrees with the expectation, since roGFP2-Orp1 functions as direct H_2_O_2_ sensor while the glutathione pool is oxidized indirectly by regenerating H_2_O_2_-oxidized PRXIIB/D through the GSH/GRX-system.

**Fig. 3:**
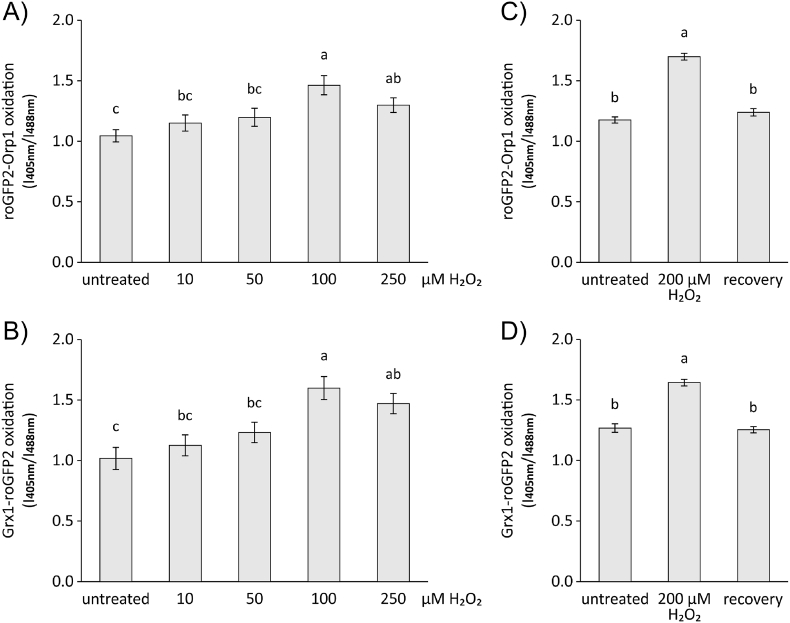
Cytosolic redox states in *A. thaliana* protoplasts. roGFP2-Orp1 (A, C) and Grx1-roGFP2 (B, D) were expressed transiently in *A. thaliana* protoplasts under control of the ubiquitin 10 promoter and the redox state of the sensors was measured under oxidizing conditions as indicated. (A, B) Untreated cells were measured first, then treated with different H_2_O_2_ concentrations (10, 50, 100 and 250 μM) for 30 min and the sensor responses were recorded again. Mean ± SE, n = 32–40. Significant differences were identified by ANOVA and Tukey-test and indicated by different letters (p < 0.05).(C, D) Recovery of the redox state. Cells were measured first untreated, then cells were treated with 200 μM H_2_O_2_ for 30 min and the sensor responses were recorded again. Recovery measurements started at 30 min after removal of H_2_O_2_ by replacing the media. Mean ± SE, n = 80–148. Significant differences were identified by ANOVA and Tukey-test and indicated by different letters (p < 0.05).

Adding higher H_2_O_2_ concentrations of 250, 500 and 1000 μM elicited a biphasic oxidation with exhaustion of the reductive power after 4 min followed by an increase to maximum oxidation within the subsequent following 5 min. Here it was noteworthy that 250 μM H_2_O_2_ saturated the system, albeit 500 μM GSH and 200 μM NADPH had been added corresponding to a theoretical H_2_O_2_ detoxification capacity of 450 μM. This indicates that the ability of the glutathione system to regenerate oxidized PRXs became less efficient if it is half oxidized which may be explained by kinetic constraints or by posttranslational modifications such as inhibitory glutathionylation of PRXII if GSSG accumulates (Dreyer et al., 2021). The system was able to reproduce the kinetics of sensor oxidation also after a repeated injection of H_2_O_2_, provided sufficient reduction equivalents were present (Suppl. Figs. S1A and B). Thus, the system remained fully functional during the detoxification cycle and the data was in agreement with *in vivo* measurements, which revealed a response of both redox sensors to variations in the redox conditions with a clear response at 100 μM H_2_O_2_ (Fig. 3A and B; Suppl. Figs. S4A and B) and showed full recovery at 30 min after removal of H_2_O_2_ (Fig. 3C and D).

The reconstituted system also detoxified tertiary butylhydroperoxide (tBOOH), whereas cumenehydroperoxide (CuOOH) oxidized the roGFP2-Orp1 sensor but reduction was delayed (Fig. 2 C, D). We observed an erratic bifurcated response with either slow recovery about 9 min after CuOOH addition or alternatively further oxidation with unpredictable patterns. Other controls were performed as well. 100 μM GSSG oxidized the Grx1-roGFP2 sensor only, while the simultaneous addition of 100 μM H_2_O_2_ and 100 μM GSSG oxidized both sensors.

### 3.1 Thiol peroxidases as sensors specifically drain electrons from the network

Omission of PRXIIB/D abolished the fast peak of glutathione oxidation and no oxidation was detected. Surprisingly, in the absence of both PRXIIB/D and GPXL2/8 a very slow and small creep toward oxidation occurred over time (Fig. 2 F). Glutathione oxidation was increased and reached a higher maximal oxidation level if GPXL2/8 were absent. This result was fully in line with the reported coupling of PRXII to the GRX/GSH-system and of GPXL to the TRX system [11]. The H_2_O_2_-induced oxidation of the roGFP2-Orp1-sensor increased in speed and maximum value in the absence of GPXL2/8 (Fig. 2 E). Unexpectedly, the sensor oxidation was slightly lower if PRXIIB/D or both thiol peroxidase systems were lacking, while the delayed maximum and increased persistence of sensor oxidation tentatively agreed with the described features of the peroxidases.

Calculating the redox rate as first proxy showed that the highest rate of roGFP2-Orp1 oxidation occurred immediately after addition of H_2_O_2_, independent on its concentration. In a converse manner, glutathione oxidation proceeded at highest rate only after exhaustion of the reductive power in the system (Suppl. Fig. S2). Therefore, such high H_2_O_2_ concentrations were excluded from further analysis.

**Fig. 4:**
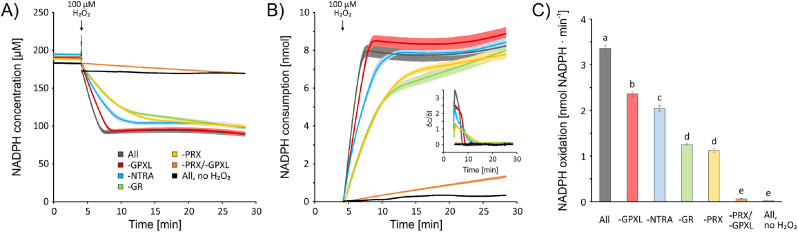
NADPH-oxidation after addition of 100 μM H_2_O_2_ in dependency on the components of the network. (A) The protein mix equilibrated with 200 μM NADPH and 500 μM GSH was measured spectrophotometrically at 340 nm. Baseline was recorded for 4 min, then 100 μM H_2_O_2_ were added to the samples in the cuvettes and OD_340_ was recorded for additional 25 min. Measurements were performed with all reconstitution components (grey), in the absence of NTRA (blue), GR (green), GPXL (red), PRX (yellow) and both, PRX and GPXL (orange). The complete reconstitution system with the addition of buffer instead of H_2_O_2_ served as control (black). (B) The NADPH-consumption was calculated based on the absorption in (A). The inserted graph in B) is derived from NADPH consumption with slopes of ten floating data points (time intervals of 8 ms) on y-axis against median of corresponding timepoints on x-axis. (C) Initial rates of NADPH-oxidation directly after addition of 100 μM H_2_O_2_ were compared for the seven conditions. Data are means ± SE with n = 8–15, except for the measurement in absence of H_2_O_2_, which has been measured once. Statistical significance was analyzed by ANOVA and Tukey post hoc test (p < 0.05). (For interpretation of the references to colour in this figure legend, the reader is referred to the Web version of this article.)

The ultimate electron donor in the cytosol for H_2_O_2_ reduction and likewise in the reconstituted network is NADPH+H^+^. We monitored NADPH+H^+^ oxidation in the reconstitution system at 340 nm (Fig. 4A–B). The graph depicts the ultimate consumption of reducing equivalents. Following addition of 100 μM H_2_O_2_ to the complete network NADPH+H^+^ was oxidized with an initial rate of 3.36 nmol min^−1^ (Fig. 4C). Omission of GPXL2/8 reduced the initial rate by 30 % (2.36 nmol min^−1^), quite similar to the exclusion of NTRA with 39 % lower rate relative to the complete assay. The difference of 9.6 % indicates that the TRX system facilitated additional pathways for H_2_O_2_ reduction, possibly via PRXIIB/D. The difference between the network lacking either GR (−63 %) or PRXII (−66 %) was not significant, but tentatively indicates the smaller contribution of the TRX system to regeneration of PRXII [11]. The very low residual rate of NADPH oxidation (1.7 %) in the absence of PRXIIB/C/D and GPXL2/8 proves that direct oxidation of other protein thiols or glutathione by H_2_O_2_ was negligible in the reconstitution system. Differences were already observed after calibration and before H_2_O_2_-addition (Suppl. Figs. S1C and D). These analyses also revealed that each pathway could only partially substitute for the lack of the other causing major kinetic changes in NADPH-oxidation (Fig. 4B), H_2_O_2_ reduction, and glutathione redox homeostasis.

### 3.2 Thiol redox proteomics proves predominant oxidation of thiol peroxidases

**Fig. 5:**
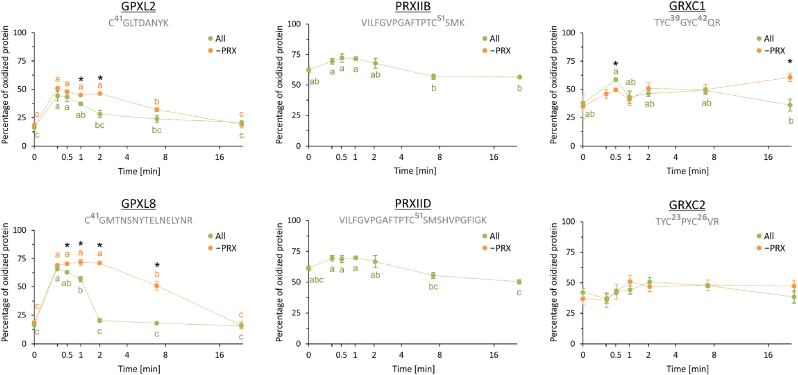
Time-dependent percentage of oxidized proteins in the reconstituted network in presence and absence of PRX after the addition of 100 μM H_2_O_2_. The Cys oxidation states of GPXL2, GPXL8, PRXIIB, PRXIID, GRXC1 and GRXC2 were determined by mass spectrometry at defined time points (0, 0.25, 0.5, 1, 2, 7 and 20 min) after H_2_O_2_ addition to the complete reconstitution system (green) or in absence of both PRXIIB and PRXIID (orange). The corresponding peptides are provided in grey letters, superscript numbers denominate the position of different cysteines. Means ± SE, n = 4. *t*-test was performed for assessing the statistical differences between samples with and without PRX (asterisks), ANOVA and Tukey post hoc test (p < 0.05) to identify significant differences between the different time points as indicated by letters. (For interpretation of the references to colour in this figure legend, the reader is referred to the Web version of this article.)

The next analysis addressed the state of the proteome in the kinetic experiment by labeling the free, reduced thiols, and the formerly oxidized ones after reduction, with light and heavy N-ethylmaleimide (NEM), respectively, followed by quantitative mass spectrometric analysis. We compared the kinetics in the presence or absence of PRXIIB/D (Fig. 5, Suppl. Fig. S3). In the complete reconstitution system, the thiols of Cys41 in GPXL2 and GPXL8, and Cys51 in PRXIIB and PRXIID oxidized within a few seconds after peroxide addition (first time point) to a variable degree. Highest oxidation was observed for GPXL8 whose oxidation state increased from about 18 % to more than 70 %. PRXIIB/D were oxidized more than GPXL2/8 before injecting H_2_O_2_ and the oxidation state only slightly increased. The GRXC1 and GRXC2 cysteine redox state was also slightly affected. Re-reduction of GPXL8 by the network was completed within 2 min indicating efficient coupling to TRXs. Re-reduction was slower in case of GPXL2, PRXIIB and PRXIID. Omission of PRXIIB/D from the system elevated the maximal oxidation state of GPXL8 and tripled the half time for its re-reduction (Fig. 5).

### 3.3 The network protects GAPC2 from oxidative inactivation

**Fig. 6:**
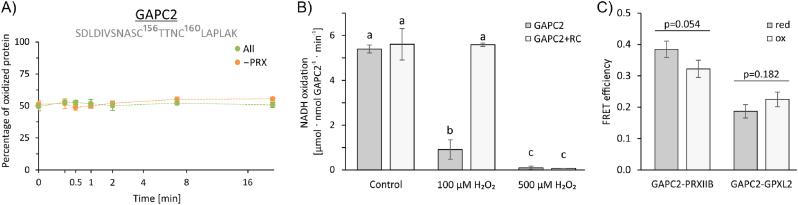
GAPC2 as target of thiol peroxidases. (A) Time-dependent percentage of oxidized GAPC2 in the reconstituted network in presence (green) and absence (orange) of PRX after addition of 100 μM H_2_O_2_. The identified peptide is given in grey letters, superscript numbers denominate the position of different cysteines. The oxidation state was determined at defined time points (as before) after H_2_O_2_-addition. Mean ± SE, n = 4. There were no significant statistical differences between samples with and without PRX (*t*-test, p < 0.05) or between the different time points (ANOVA and Tukey post hoc test, p < 0.05) (B) The GAPC2 activity was measured as change in absorbance at 340 nm corresponding to the NADH concentration. Measurements were performed in absence (grey bars) and presence (white bars) of the reconstitution components (RC) and as a control, activity was also measured for the protein mix of the reconstituted system missing GAPC2, which showed no activity. All samples contained 500 μM GSH and 200 μM NADPH. The inhibitory effect of 100 or 500 μM H_2_O_2_ was analyzed after 30 min incubation. Means ± SD, n = 6. Statistical significance was analyzed by ANOVA and Tukey post hoc test (p < 0.05). (C) *In vivo* FRET-measurements between GAPC2 and the thiol peroxidases in protoplasts. mTurquoise2 serving as donor and eYFP as acceptor were fused to GAPC2 and the thiol peroxidases, respectively, and transiently expressed under control of the CaMV35S-promoter. FRET-efficiency was determined by sensitized acceptor emission. As indicated, cells were reduced by 1 mM DTT or reduced cells were oxidized by 5 mM H_2_O_2_. Mean ± SE, n = 23–37. *t*-test was performed to identify significant differences. (For interpretation of the references to colour in this figure legend, the reader is referred to the Web version of this article.)

The reconstitution assay contained two redox target proteins, the cytosolic malate dehydrogenase 1 (MDH1) and the glyceraldehyde-3-phosphate dehydrogenase GAPC2. The redox state of the relevant thiols of Cys 156 and 160 of GAPC2 remained unchanged upon addition of 100 μM H_2_O_2_ to the complete reconstitution system (Fig. 6A), whereas the same H_2_O_2_ spike inhibited GAPC2 by 84 % in the absence of the network. 500 μM H_2_O_2_ caused complete inhibition (Fig. 6B). Apparently, the complete system fully protected GAPC2 from oxidative inactivation or regenerated the oxidized form by redox transmitters.

The possible interaction between GAPC2 and either PRXIIB or GPXL2 was studied by fluorescence resonance energy transfer (FRET) in transfected protoplasts from *A. thaliana* leaves (Fig. 6C; Suppl. Fig. S4C). Under reducing conditions FRET efficiency reached a value of 0.39. This value decreased significantly to 0.32 upon treatment with H_2_O_2_. FRET efficiency was about halved for the system of GAPC2 and GPXL2. The second target protein was MDH1 (Suppl. Figs. S4C and S5). However, MDH1 was neither oxidized in the network context nor as isolated protein. Interestingly, FRET showed significant interaction of MDH1 both with GPXL2 and PRXIIB.

### 3.4 Ascorbate peroxidase lowers but cannot abolish sensor oxidation

**Fig. 7:**
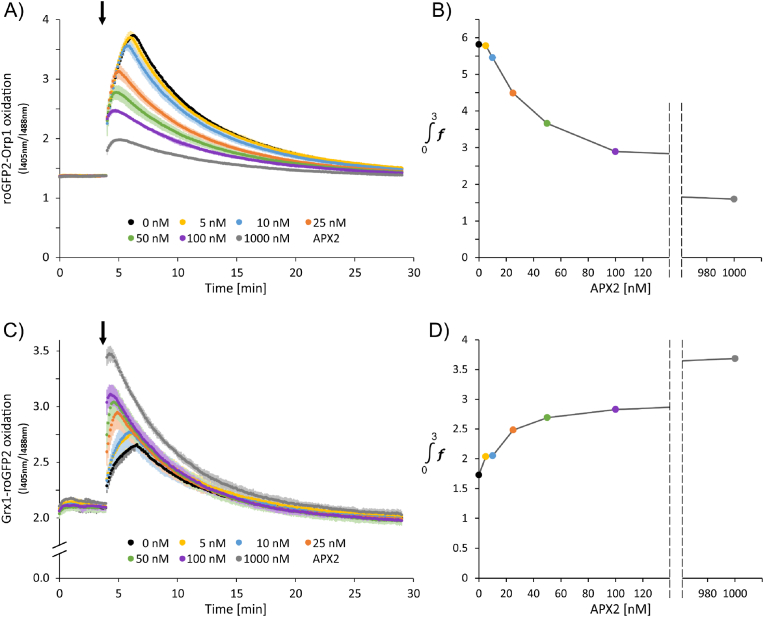
Changes in H_2_O_2_ concentration and E[GSH] in the reconstituted network supplemented with APX2. The fully reconstituted system contained APX as indicated and in addition 1.88 μM DHAR1 and 1 mM ASC. H_2_O_2_ and E[GSH] were monitored with roGFP-Orp1 or Grx1-roGFP. GSH and NADPH concentrations were elevated to 1 mM both. Oxidation state was measured by laser scanning microscopy as ratio of the fluorescence emissions at the excitation wavelengths 405 nm and 488 nm. The emissions were recorded for 30 min. The first 4 min show baseline recordings of the equilibrated system, then 100 μM H_2_O_2_ was added (arrow). (A) Response of roGFP2-Orp1 to increasing APX2 concentrations. (B) Given data is derived from A) and shows the area under each individual peak, for only initial 3 min after H_2_O_2_ addition. For area calculation, the product of summarized data points and the total time i.e., 3 min was divided by number of total data points (n = 31) in the same time interval. The data point values were normalized against baseline before area calculations. C) Response of Grx1-roGFP2 to increasing APX2 concentrations. Mean ± SD is shown, n = 2–3. D) derived from C) and has been calculated as above.

Ascorbate peroxidases (APX) are efficient H_2_O_2_ reductases in various plant cell compartments including the cytosol. Ascorbate is the primary electron donor, however, dehydroascorbate reductase (DHAR) reduces dehydroascorbate to ascorbate at the expense of glutathione that is oxidized to GSSG. This reaction couples APX-dependent H_2_O_2_ reduction to the glutathione system and NADPH via GR. The effect of APX supplementation on the network's performance was addressed in an enzyme concentration series ranging from 5 nM to 1 μM APX2 with 1.88 μM DHAR1 and 1 mM ASC (Fig. 7). With the previously used levels of 500 μM GSH and 200 μM NADPH, fluorescent sensors were still transiently oxidized after addition of 100 μM H_2_O_2_. In presence of increased concentrations of 1 mM GSH and 1 mM NADPH, the magnitude of roGFP2-Orp1 sensor oxidation in the network decreased with increasing APX activity (Fig. 7A), quantifiable as mathematical integral of the curve during the first 2 min after H_2_O_2_ addition (Fig. 7B). Inversely, enhanced Grx1-roGFP2 oxidation revealed immediate oxidation of GSH by DHAR and transient accumulation of GSSG (Fig. 7C and D). This result indicates that the presence of APX cannot protect the network from sensing and responding to the H_2_O_2_ stimulus, but significantly shifts the oxidative burden to the glutathione pool.

## 4 Discussion

### 4.1 Redox bursts are common to strong environmental switches

This work demonstrates successful and stable reconstitution of the central part of the cytosolic redox regulatory network. The cytosol is an integrator of ROS signaling in plants that determines the redox state of many proteins and thereby metabolism, acclimatization, and cell fate. When subjected to rapid and severe changes in the abiotic or biotic environment, e.g. high temperature, excess photosynthetic active radiation (PAR), pathogen infection, re-aeration after hypoxia, or osmotic shock [[36], [37], [38], [39]], cells respond with increased ROS generation that is immediately linked to changes in cytosolic H_2_O_2_. E.g., up-shifts in PAR initiate local and systemic signaling linked to O_2_^.-^ and H_2_O_2_ [40]. H_2_O_2_ released from the photosynthesizing or photodamaged chloroplast to the cytosol serves as retrograde signal to control multiple metabolic adjustments [41].

The inter-compartmental transfer of H_2_O_2_ signals was demonstrated by Ugalde et al. [42] in seedlings expressing roGFP-Orp1 or Grx1-roGFP, the gene-encoded sensors also used here in the reconstitution system. Ugalde et al. applied a combination of methylviologen, light and the photosynthetic inhibitor 3-(3,4-dichlorophenyl)-1,1-dimethylurea (DCMU) and revealed ROS- and glutathione-related signal transfer from the chloroplast to the cytosol and mitochondrion. Apart from the photosynthetic electron transport chain in the chloroplast other generator systems for O_2_^.-^ and H_2_O_2_ are the respiratory electron transport chain in the mitochondrion, the peroxisomes with their highly active oxidase-dependent metabolism and the Respiratory Burst Oxidase Homologue (RBOH) in the plasma membrane [43]. H_2_O_2_ escapes from the organelles and reaches the cytosol. This integrative function of the cytosol in H_2_O_2_ signaling prompted us to explore the capacity and dynamics of the cytosolic redox regulatory network and we administered H_2_O_2_ to the system for investigating its wiring, capacity, and dynamics.

### 4.2 The H_2_O_2_ spike is in the physiological range

We opted for a single H_2_O_2_ spike of 100 μM H_2_O_2_ (Fig. 1, Suppl. Fig. S1). How does this H_2_O_2_ amount compare with electron flow in PET in leaves? A typical CO_2_ assimilation rate in ambient CO_2_ and saturating PAR is 100 μmol CO_2_^.^mg chlorophyll^−1.^h^−1^ equivalent to 400 μmol electrons^.^mg chlorophyll^−1.^h^−1^ used in photochemistry [44]. In fact, the rate of PET likely is higher, since a major portion of PET activity is not preserved in carbon assimilation due to photorespiration, alternative electron acceptors or dissipation mechanisms. The cytosol of the photosynthetic mesophyll occupies between 5 and 10 % of the mesophyll cell volume, while the chloroplasts fill between 25 and 30 % [45,46]. 12^.^10^6^ mesophyll cells^.^mg chlorophyll^−1^ in barley have a total volume of 379 μl [45]. Given these numbers 100 μM H_2_O_2_ corresponds to 1.5^.^10^15^ H_2_O_2_ molecules administered to the cytosol volume equivalent to 1 mg chlorophyll. If all electrons used for carbon fixation would be diverted to synthesize H_2_O_2_, the H_2_O_2_ amount would be > 8000 times higher per hour than the amount injected and assuming a 1% use of electrons in PET for H_2_O_2_ synthesis, it would take less than 45 s to generate the amount injected in our reconstituted system.

Based on literature data, Polle (2001) modelled the water-water cycle of the chloroplasts with a rate of 240 μM O_2_^.-.^s^−1^ and a rate of 120 μM H_2_O_2_ s^−1^ [47,48]. Given the high activity of SOD and the high surface area of chloroplasts, that is in the range of the area of the plasma membrane providing enough contact site for releasing this amount of H_2_O_2_ from the chloroplast to the cytosol [41], we conclude that the administered H_2_O_2_ amount was well in the physiological range occurring in light shift or stress experiments.

It would be interesting to also consider RBOH as source of H_2_O_2_ and compare its rate with the given H_2_O_2_ amount, however we could not find appropriate stoichiometric data, since most RBOH activity determinations use XTT (3'-[1-[phenylaminocarbonyl]-3,4-tetrazolium]-bis(4-methoxy-6-nitro)benzenesulfonic acid) to assess O_2_^.-^ generation semi-quantitatively.

### 4.3 The reconstituted network is robust

This work combines network reconstitution from recombinant proteins at relevant physiological concentrations with response monitoring of H_2_O_2_ and [GSH]^2^/GSSG-redox sensors and thiol redox proteomics as reliable read-out. This novel and unique approach enabled studying network dynamics and performance. Recently used assays for enzyme activity assessment often used dithiothreitol or heterologous redox transmitters as electron donors [49,50], or established single electron transfer pathways from NADPH to H_2_O_2_ [11]. The robustness of the fully reconstituted system of this study was proven by showing that the enzyme stability was not limited but the availability of reductant. Addition of extra NADPH enabled reproducing the H_2_O_2_-induced oxidation and reduction kinetics of the sensors without hysteresis (Suppl. Fig. S1). Thus, H_2_O_2_ was a mild and not damaging oxidant in this redox network, while addition of the bulky CuOOH (Fig. 2C and D) caused modifications to the kinetics indicating more severe oxidation compared to H_2_O_2_. This has been observed for PRXIIE where low amounts of CuOOH caused irreversible sulfinic and sulfonic acid formation [51]. However, CuOOH is an artificial peroxide substrate and similarly bulky alkylhydroperoxides unlikely occur at similar concentrations *in vivo*. In addition, the conformations and activities of the proteins were stable in the reconstituted network after H_2_O_2_-addition (Suppl. Fig. S6). It is concluded that the reconstitution system is a unique and robust network to query the open questions concerning, e.g., the connectivity of redox-active proteins upon an H_2_O_2_ burst.

### 4.4 Thiol and ascorbate peroxidases balance H_2_O_2_ detoxification

The coupling of GPXL-dependent H_2_O_2_ reduction to the TRX system and of PRX activity predominantly to the GRX/GSH system indicates division of labor in H_2_O_2_ detoxification but also differences in downstream signal transduction. The concept of shared burden is further supported if also considering APX, which is primarily linked to the ascorbate pool. Ascorbate concentrations in the cytosol of Arabidopsis and barley, measured with fractionation methods or immunological approaches, range between 20 and 35 mM [52,53]. This concentration exceeds the cytosolic glutathione concentrations reported with 3.5 mM 6- to 10-fold [54]. We examined the possibility to increase the ascorbate concentration in the reconstitution system to 30 mM with the intention to omit DHAR and to uncouple APX dependent H_2_O_2_ reduction from the NADPH pool. However, ascorbate at that concentration continuously oxidized the NADPH pool in the system even without addition of H_2_O_2_ at a rate of 0.36 nmol NADPH^.^min^−1^. Therefore, we did follow up on this concept.

Cytosolic NADPH concentrations are in the range of 150 μM [55]. Thus, the ratio of redox buffer capacity present in the pools of NADPH: GSH: ascorbate is about 1 : 23 : 133, suggesting that exclusively using the APX pathway might be most efficient in detoxifying cytosolic H_2_O_2_. The presence of the thiol peroxidases indicates a high significance of PRX- and GPXL-dependent processes in tuning H_2_O_2_ concentrations for defense and proper cell signaling in a kinetic, spatial, or network-coupling specific manner different to APX.

In addition to the low molecular mass redox buffers, we have to consider the protein amounts and the affinities to H_2_O_2_. The proteome study used for choosing the concentrations for network reconstitution (Fig. 1), also detected cytosolic APX1, while APX2 and 6 were below the detection limit. The estimated cytosolic protein abundance of APX1: PRXII: GPXL and their ratio were 1 : 0.30: 0.13. Since the K_M_-value of APX for ascorbate is in the range of 200–400 μM [56], the reductant as co-substrate is always saturating. Consequently, the efficiency of H_2_O_2_ detoxification by APX and the adjustment of the resting cytosolic H_2_O_2_ concentration depend on its activation state, e.g. by posttranslational modifications, conditional gene expression and protein synthesis.

### 4.5 Network interaction with ascorbate peroxidase

*vtc* mutants with a limitation in ascorbate synthesis have less than 30 % ascorbate of the wild type level. Using these plants, Plumb and co-workers demonstrated that ascorbate is needed for proper growth of *A. thaliana*, but less for photoprotection of photosynthesis, despite the fact that ascorbate amounts in wild type plants doubled after transfer to 6-fold higher PAR, while the ascorbate amounts remained unchanged in *vtc2-1* and *vtc2-4* lines [57]. APX-catalyzed ascorbate-dependent H_2_O_2_ reduction activity is inhibited when APX is preincubated with either the NTR-system or glutathione [58]. The proposed mechanism involves the transiently formed thiyl radical reacting with thiyl anions and O_2_ producing H_2_O_2_ [59]. Catalase and PRXII prevent this inactivation [58]. Likewise, APX loses its activity if incubated with H_2_O_2_ in the absence of ascorbate [47]. APX activity is also regulated by S-nitrosylation, tyrosine nitration, and S-sulfhydration [60,61]. S-nitrosylation stimulates APX1 activity, lowering H_2_O_2_ levels and increasing stress sensitivity [62]. These reports suggest that detoxification of H_2_O_2_ through the APX pathway is regulated and subject to stress-dependent inactivation. Under such conditions, the thiol redox regulatory network may gain importance in H_2_O_2_ reduction beyond signaling.

### 4.6 Signaling function of thiol peroxidases

The discussed facts call for a revision of the network wiring. Cytosolic APX assists in adjusting the low resting H_2_O_2_ concentration. By its tight linkage via DHAR to the glutathione pool, APX can alter the glutathione redox state upon a H_2_O_2_ burst (Fig. 7). The ascorbate buffering capacity is extremely high as discussed above. Thiol peroxidases display slightly higher H_2_O_2_ affinities than APX, with PRXs having very low pK (Cys-SH) and high catalytic efficiencies. The human homologue of cytosolic type II PRX, PRDX5 displays a pK = 4.6 for thiolate formation at the peroxidatic Cys47 and a catalytic efficiency k_app_ (H_2_O_2_) = 4.3^.^10^5^ M^−1^ s^−1^ [63] and thus, 232-fold less efficient than PRDX2 with an k_app_ = 10^8^ M^−1^ s^−1^ [64] but still many orders of magnitude higher than cysteinyl thiols lacking the catalytic center environment of thiol peroxidases. The apparently lower catalytic efficiency originates from the complex reduction mechanism involved in regenerating reduced and active PRX and GPXL. K_M_ (H_2_O_2_) values for PRXs of below 1 μM have been reported from stop flow approaches, but interpretation of such data is difficult in the light of the regeneration limitation [65]. The K_M_ (H_2_O_2_) of APX is estimated to be around 3 μM [66]. Only competitive assays under physiological condition such as those established in the reconstitution system provide reliable access to the ability of protein thiols in the network to be oxidized in the presence of APX. The titration experiment with increasing APX2 amounts added to the reconstituted network demonstrated that oxidation of the Orp1-sensor representative for PRXs still occurred very fast, but with lower magnitude, upon injection of H_2_O_2_ in the presence of APX (Fig. 7). The oxidation of thiol peroxidases in the presence of APX allows for redox signal transfer from PRX and GPXL to redox regulated target proteins using different mechanisms [67].

### 4.7 The thiol redox-regulatory network efficiently protects protein thiols from oxidation

The proteomic analysis demonstrates the strong transient oxidation of the catalytic GPXL thiol, in particular of GPXL8 whose oxidation state increased from 16 % to 67 % after H_2_O_2_ addition (Fig. 5), while catalytic thiols of PRX and GPXL2 were less affected and all other detected protein thiols remained unchanged (Fig. 5, Suppl. Fig. S3). The presence of the network protected GAPC2 from being oxidized and inhibited after addition of H_2_O_2_ (Fig. 6), whereas GAPC2 was inhibited *in vitro* in the absence of the network by H_2_O_2_ to 16 % residual activity. GAPC1 and GAPC2 are the two phosphorylating isoforms of glyceraldehyde-3-phosphate dehydrogenase in the cytosol that function in glycolytic metabolism and in addition display moonlighting functions in gene expression control and posttranslational regulation as RNA binding protein [[68], [69], [70]]. Imaging of GAPC in living cells documented preferred association with mitochondria under reducing conditions and translocation to the nucleus under oxidizing conditions [71]. Maintenance of reduced GAPC2 in the reconstitution systems upon H_2_O_2_ supplementation indicates that the capacity of the redox network is sufficient to detoxify the H_2_O_2_ and to avoid GAPC1/2 oxidation. In part this may also be owed to the presence of TRXh isoforms that can reduce oxidized GAPC [71] counteracting slow accumulation of oxidized GAPC. The TRX-reduction pathway coupled to NTRA and NADPH likely efficiently regenerated TRXh3 primarily feeding electrons into the GPXL pathway.

### 4.8 Toward identifying targets of thiol redox network-dependent signaling

Several biochemical mechanisms allow for linking the thiol redox state of the network with the activity and function of redox target proteins such as transcription factors or metabolic enzymes [67,72]. APX-dependent H_2_O_2_ detoxification circumvents the protein thiol network but oxidizes the glutathione pool during DHAR-dependent reduction of dehydroascorbate (Fig. 7). Already low amounts of GSSG foster glutathionylation of thiols in redox target proteins and alter their function [18]. PRXIIE and GAPC are examples of target proteins that undergo glutathionylation if GSSG levels increase [19,51].

But even in the absence of APX, the presence of PRXIIB/C/D is sufficient to catalyze transient glutathione oxidation as indicated by the Grx1-roGFP2-sensor (Fig. 2F). Thus, both APX and PRXII could promote glutathionylation upon exposure to H_2_O_2_. Glutathione remained reduced in the absence of APX and PRX.

Upon reaction with H_2_O_2_, the peroxidatic cysteinyl sulfur of thiol peroxidases adopts the sulfenic acid form. Its subsequent reaction with suitable thiols of target proteins establishes at least intermittently an intermolecular disulfide bridge and can alter function of target proteins [73]. The protein phosphatase Abscisic Acid Insensitive 2 (ABI2) was shown to form such intermediates with GPXL and PRXII [74,75], and this process is important to trigger stomatal closure upon pathogen attack. Intermolecular disulfide formation appears unexplored for cytosolic thiol peroxidases in plants, although *in vitro* capturing of PRX interactors has successfully been conducted for the chloroplast 2-CysPRX and PRXIIE and allowed for separating salt-displaceable from redox-dependently bound interactors [51,76]. The high similarity between the chloroplast PRXIIE and the cytosolic PRXIIB/C/D may explain, why the analysis of the binding partners of PRXIIE identified many cytosolic proteins, in particular various 14-3-3 proteins [51].

GPXL are regenerated by the redox transmitters TRXs [11]. Oxidized redox transmitters in turn can function as oxidants of target proteins as shown for the regulation of the Calvin Benson cycle [[77], [78], [79]]. The participation of this mechanism in redox signaling in the cytosol should be explored in more detail. Local microdomain formation between targets, redox transmitters and redox sensors may facilitate this type of regulation. The transient accumulation of oxidized GPXL2/8 after H_2_O_2_ injection reveals kinetic limitations of TRX-dependent regeneration. The specific coupling to the TRX system [11] distinguishes GPXL2/8 reduction from PRXIIB/C/D reduction. It is tempting to speculate that these different branches in the network interact with distinct target proteins and might explain, why in plants TRX-dependent PRXs are absent from the cytosol in contrast to animals and fungi.

## 5 Conclusion

The study provides unique insight into the wiring of the functional elements in the redox regulatory network of the plant cytosol. The here introduced reconstitution system of the redox regulatory network offers the novel opportunity to test candidates from proteome studies under defined and stoichiometric conditions. Reconstituting the thiol redox regulatory network of the cytosol *in vitro* also allows for dissecting the contribution of the network elements and the functional coupling of its pathways during the response to an H_2_O_2_ pulse simulating an H_2_O_2_ burst during stress-induced cell signaling and stress acclimatization. The simultaneous monitoring of H_2_O_2_, [GSH]2/[GSSG] redox potential and NADPH+H^+^ revealed branching of the electron transfer within the system with significance for cell signaling and the role of ascorbate peroxidase in tuning the pathway dynamics. This advanced test system bridges the large and challenging gap between oversimplified *in vitro* tests and genetic approaches that suffer from the severe drawbacks of redundancy and compensatory mechanisms in biology, particularly in redox regulation in plants.

## Funding

This work was funded by the 10.13039/501100001659Deutsche Forschungsgemeinschaft (DFG, German Research Foundation) - project numbers: 496357082, 469950637 (IF) and DI 346/23 (KJD).

## CRediT authorship contribution statement

**Lara Vogelsang:** Writing – review & editing, Writing – original draft, Validation, Methodology, Investigation, Formal analysis, Data curation, Conceptualization. **Jürgen Eirich:** Investigation, Formal analysis, Data curation. **Iris Finkemeier:** Writing – review & editing, Supervision, Resources, Project administration, Funding acquisition, Conceptualization. **Karl-Josef Dietz:** Writing – review & editing, Writing – original draft, Supervision, Resources, Project administration, Funding acquisition, Conceptualization.

## Declaration of competing interest

The authors declare that they have no known competing financial interests or personal relationships that could have appeared to influence the work reported in this paper.

## Data Availability

Data will be made available on request. The proteome data have been deposited at https://repository.jpostdb.org/preview/171621217365fbea558369b.
