# Supplementary material for: Specificity and dynamics of H2O2 detoxification by the cytosolic redox regulatory network as revealed by in vitro reconstitution
Source: Redox Biol. 2024 Apr 3;72:103141. doi: 10.1016/j.redox.2024.103141 (PMC11022108; doi:10.1016/j.redox.2024.103141)
Supplement: Multimedia component 1 [file mmc1.pdf]

Supplemental Materials: Vogelsang et al.

Suppl. Table S1: Abbreviations, Arabidopsis genome identifiers (AGI) and functions of used and mentioned proteins.

		**AGI**	**Function**
PRXIIB	Peroxiredoxin type II B	At1g65980	Sensor
PRXIIC	Peroxiredoxin type II C	At1g65970	Sensor
PRXIID	Peroxiredoxin type II D	At1g60740	Sensor
GPXL2	Glutathione peroxidase-like 2	At2g31570	Sensor
GPXL8	Glutathione peroxidase-like 8	At1g63460	Sensor
TRXh1	Thioredoxin h1	At3g51030	Transmitter
TRXh2	Thioredoxin h2	At5g39950	Transmitter
TRXh3	Thioredoxin h3	At5g42980	Transmitter
TRXh4	Thioredoxin h4	At1g19730	Transmitter
TRXh5	Thioredoxin h5	At1g45145	Transmitter
TRXh7	Thioredoxin h7	At1g59730	Transmitter
TRXh8	Thioredoxin h8	At1g69880	Transmitter
TRXh9	Thioredoxin h9	At3g08710	Transmitter
TDX	TPR repeat-containing thioredoxin	At3g17880	Transmitter
GRXC1	Glutaredoxin C1	At5g63030	Transmitter
GRXC2	Glutaredoxin C2	At5g40370	Transmitter
NTRA	NADPH-dependent thioredoxin reductase A	At2g17420	Input element
NTRB	NADPH-dependent thioredoxin reductase B	At4g35460	Input element
GR	Glutathione reductase	At3g24170	Input element
MDH1	Malate dehydrogenase 1	At1g13440	Target
GAPC2	Cytosolic NAD-dependent glyceraldehyde-3-phosphate dehydrogenase C subunit 2	At1g13440	Target
APX1	Ascorbate peroxidase 1	At1g07890	Sensor
APX2	Ascorbate peroxidase 2	At3g09640	Sensor
DHAR1	Dehydroascorbate reductase 1	At1g19570	
DHAR2	Dehydroascorbate reductase 2	At1g75270	
PGK3	Phosphoglycerate kinase 3	At1g79550	

Suppl. Table S2: List of nucleotide primers used for molecular cloning.

	AGI	Vector	Restriction enzyme		Primer Sequence 5'->3'
PRXIIB[1]	At1g65980	pET28a	NdeI	Forward	AAAACATATGGCTCCAATTGCTGTCGGCGATG
			BamHI	Reverse	TTTTGGATCCTTAGAGAGCCTTGAGGATATCATCGGCGCTG
PRXIID[2]	At1g60740	pET28a	NdeI	Forward	AAAACATATGGCTCCAATTACTGTCGGCGATG
			BamHI	Reverse	TTTTGGATCCTTAGAGAGCCTTAAGGATATCCTCTGCGCTG
GPXL2[2]	At2g31570	pET28a	NdeI	Forward	AAAACATATGGCGGATGAATCTCCAAAGTCTATC
			BamHI	Reverse	TTTTGGATCCTTAAGAAGAGGCCTGTCCCAACG
GPXL8[1]	At1g63460	pET28a	NdeI	Forward	AAAACATATGGCGACGAAGGAACCAG
			XhoI	Reverse	TTTTCTCGAGTCAGGAGATATTCAGAAGATTCTTTATGTC
TRXh1[2]	At3g51030	pET28a	NdeI	Forward	AAAACATATGGCTTCGGAAGAAGGACAAGTG
			BamHI	Reverse	TTTTGGATCCTTAAGCCAAGTGTTTGGCAATGGTAGAC
TRXh2[2]	At5g39950	pET28a	NdeI	Forward	AAAACATATGGGAGGAGCTTTATCAAC
			XhoI	Reverse	TTTTCTCGAGTTATGCTCTGAGTTTGCT
TRXh3[1]	At5g42980	pET28a	NdeI	Forward	AAAACATATGGCCGCAGAAGGAGAAGTTATC
			BamHI	Reverse	TTTTGGATCCTCAAGCAGCAGCAACAACTGTCTTG
TRXh5[1]	At1g45145	pET28a	NdeI	Forward	AAAACATATGGCCGGTGAAGGAGAA
			XhoI	Reverse	TTTTCTCGAGTCAAGCAGAAGCTACAAGAC
TDX[2]	At3g17880	pET28a	NheI	Forward	AAAAGCTAGCATGGTGGATGCGATTCAAGTAG
			BamHI	Reverse	TTTTGGATCCTTACTTAGAAGAAGAGTGCTGTGCAATC
GRXC1[2]	At5g63030	pET28a	NheI	Forward	AAAAGCTAGCATGGGTTCAATGTTCAGTGGAAAC
			BamHI	Reverse	TTTTGGATCCTCAAAGTTGAGAAGAGTTATCTGCAATAGC
GRXC2[1]	At5g40370	pET28a	NheI	Forward	AAAAGCTAGCATGGCGATGCAGAAAGCTAAG
			BamHI	Reverse	TTTTGGATCCTTAAGCAGAAGTTGTTGCAGTCTTTC
NTRA[1]	At2g17420	pET28a	NdeI	Forward	AAAACATATGTCCTCCGCCGCCGCCGCCGT
			BamHI	Reverse	TTTTGGATCCTCAATCACTCTTACCCTCCTGAGATCCAATCTCTTG
GR[1]	At3g24170	pET28a	NheI	Forward	AAAAGCTAGCATGGCGAGGAAGATGCTTG
			BamHI	Reverse	TTTTGGATCCTCATAGATTTGTCTTAGGTTTGGGTTTGTG
PGK3[1]	At1g79550	pET28a	NheI	Forward	AAAACATATGGCGACGAAGAGAAGCGTTG
			BamHI	Reverse	TTTTCTCGAGTCAAGCTTCGTCGAGAGCGAG
MDH1[3]	At1g13440	pET16b	NdeI	Forward	GGGAATTCCATATGGCGAAAGAACCAGTTCGTG
			BamHI	Reverse	CGGGGATCCTTAAGAGAGGCATGAGTACGCC
GAPC2[4]	At1g13440	pET16b	NdeI	Forward	GCGCGCCATATGGCTGACAAGAAGATTAG
			BamHI	Reverse	GCGCGCGGATAATTAGGCCTTTGACATGTG
APX2	At3g09640	pET28a	BamHI	Forward	AAAAGGATCCATGGTGAAGAAGAGTTACCCGGAAG
			NotI	Reverse	TTTTGCGGCCGCTTACTCCTTGTCAGCAAACCCGAG
DHAR1	At1g19570	pET28a	NheI	Forward	AAAAGCTAGCATGGCTCTGGAAATCTGTG
			EcoRI	Reverse	TTTTGAATTCTCAAGGGTTAACCTTGGGAG
pUBI10	At4g05320	35S-eYFP	HindIII	Forward	AAAAAAGCTTTACCCGACGAGTCAGTAA

			BamHI	Reverse	TTTTGGATCCAGTGTTAATCAGAAAAACTC
roGFP2-Orp1	-	UBI10-eYFP	NcoI	Forward	AAAACCATGGTGAGCAAGGGCGAGG
			NotI	Reverse	AAAAGCGGCCGCCTATTCCACCTCTTTCAAAAGTTCTTCGATG
Grx1-roGFP2	-	UBI10-eYFP	NcoI	Forward	AAAACCATGGCTCAAGAGTTTGTGAACTGC
			NotI	Reverse	AAAAGCGGCCGCTTACTTGTACAGCTCGTCCATGC
PRXIIB	At1g65980	35S-YFP/mT-NosT	BamHI	Forward	AAAAGGATCCATGGCTCCAATTGCTGTCG
			AgeI	Reverse	TTTTACCGGTCCGAGAGCCTTGAGGATATCATCGG
GPXL2	At2g31570	35S-YFP/mT-NosT	BamHI	Forward	AAAAGGATCCATGGCGGATGAATCTCCAAAGTC
			AgeI	Reverse	TTTTACCGGTCCAGAAGAGGCCTGTCCCAAC
MDH1	At1g13440	35S-YFP/mT-NosT	BamHI	Forward	AAAAGGATCCATGGCGAAAGAACCAGTTCGTGTG
			AgeI	Reverse	TTTTACCGGTCCAGAGAGGCATGAGTACGCCAAG
GAPC2	At1g13440	35S-YFP/mT-NosT	BamHI	Forward	AAAAGGATCCATGGCTGACAAGAAGATTAGAATCG
			AgeI	Reverse	TTTTACCGGTCCGGCCTTTGACATGTGAACGATAAG

[1]Knieper, M., Vogelsang, L., Guntelmann, T., Sproß, J., Gröger, H., Viehhauser, A., & Dietz, K. J. (2022). OPDAylation of thiols of the redox regulatory network in vitro. Antioxidants, 11(5), 855.

[2]Vogelsang, L., & Dietz, K. J. (2023). Regeneration of cytosolic thiol peroxidases. Physiologia Plantarum, 175(5), e14042.

[3]Liszka, A., Schimpf, R., Cartuche Zaruma, K. I., Buhr, A., Seidel, T., Walter, S., ... & Selinski, J. (2020). Three cytosolic NAD-malate dehydrogenase isoforms of Arabidopsis thaliana: on the crossroad between energy fluxes and redox signaling. Biochemical Journal, 477(19), 3673-3693.

[4]Holtgrefe, S., Gohlke, J., Starmann, J., Druce, S., Klocke, S., Altmann, B., Wojtera, J., Lindermayr, C. & Scheibe, R. (2008). Regulation of plant cytosolic glyceraldehyde 3-phosphate dehydrogenase isoforms by thiol modifications. Physiologia plantarum, 133(2), 211-228.

Suppl. Table S3: List of proteins and expression strains, respectively.

Protein	Expression strain	Protein	Expression strain
PRXIIB	NiCo21(DE3)	GRXC2	NiCo21(DE3)
PRXIID	NiCo21(DE3)	NTRA	NiCo21(DE3)
GPXL2	Rosetta-gami 2(DE3)pLysS	GR	NiCo21(DE3)
GPXL8	NiCo21(DE3)	PGK3	NiCo21(DE3)
TRXh1	NiCo21(DE3)	MDH1	BL21 (DE3) pLysS
TRXh2	NiCo21(DE3)	GAPC2	NiCo21(DE3)
TRXh3	NiCo21(DE3)	APX2	NiCo21(DE3)
TRXh5	NiCo21(DE3)	DHAR1	NiCo21(DE3)
TDX	Rosetta-gami 2(DE3)pLysS	roGFP2-Orp1	BL21 (DE3) pLysS
GRXC1	Rosetta-gami 2(DE3)pLysS	Grx1-roGFP2	NiCo21(DE3)

Suppl. Table S4: iBAQ-values of the proteins from four different samples/treated plants. Data has been deposited here: URL: https://repository.jpostdb.org/preview/171621217365fbea558369b Access key: 3173

		Control		Arsenic		Hypoxia		Arsenic+Hypoxia		Mean	
		iBAQ	Part of total protein	iBAQ	Part of total protein	iBAQ	Part of total protein	iBAQ	Part of total protein		Part of total protein
At2g31570	**GPXL2**	1.25E+08	0.025%	2.63E+08	0.061%	1.34E+08	0.026%	1.53E+08	0.032%	**GPX2**	0.036%
At1g63460	**GPXL8**	1.65E+07	0.003%	4.09E+07	0.009%	2.43E+07	0.005%	3.21E+07	0.007%	**GPX8**	0.006%
At2g17420	**NTRA**	5.92E+07	0.012%	5.68E+07	0.013%	1.33E+08	0.025%	7.42E+07	0.016%	**NTRA**	0.017%
At3g24170	**GR**	2.87E+07	0.006%	2.86E+07	0.007%	4.83E+07	0.009%	5.95E+07	0.013%	**GR**	0.009%
At3g51030	**TRXh1**	1.18E+06	0.0002%	4.40E+05	0.0001%	2.00E+06	0.0004%	4.82E+05	0.0001%	**TRXh1**	0.0002%
At5g39950	**TRXh2**	2.14E+07	0.004%	2.22E+07	0.005%	1.11E+07	0.002%	9.51E+06	0.002%	**TRXh2**	0.003%
At5g42980	**TRXh3**	5.88E+08	0.120%	8.45E+08	0.196%	1.01E+09	0.193%	7.30E+08	0.155%	**TRXh3**	0.166%
At1g45145	**TRXh5**	1.33E+08	0.027%	6.61E+08	0.154%	2.81E+08	0.054%	3.03E+08	0.064%	**TRXh5**	0.075%
At5g63030	**GRXC1**	2.30E+07	0.005%	4.90E+07	0.011%	3.14E+07	0.006%	3.68E+07	0.008%	**GRXC1**	0.007%
At5g40370	**GRXC2**	3.61E+08	0.074%	5.85E+08	0.136%	5.18E+08	0.099%	5.59E+08	0.119%	**GRXC2**	0.107%
At1g65980	**PRXIIB**	4.53E+08	0.092%	3.75E+08	0.087%	5.63E+08	0.108%	5.14E+08	0.109%	**PRXIIB**	0.099%
At1g60740	**PRXIID**	-	-	9.36E+05	0.0002%	1.26E+06	0.0002%	1.31E+05	0.00003%	**PRXIID**	0.0002%
At3g17880	**TDX**	2.23E+06	0.0005%	5.36E+06	0.001%	9.29E+06	0.002%	1.08E+07	0.002%	**TDX**	0.001%
At1g13440	**GAPC2**	3.26E+09	0.664%	3.21E+09	0.746%	4.41E+09	0.843%	4.00E+09	0.851%	**GAPC2**	0.776%
At1g04410	**MDH1**	1.26E+09	0.256%	1.33E+09	0.309%	1.36E+09	0.259%	1.15E+09	0.245%	**MDH1**	0.267%
At1g19570	**DHAR1**	3.99E+08	0.081%	6.76E+08	0.157%	1.33E+09	0.255%	1.37E+09	0.292%	**DHAR1**	0.196%
At1g75270	**DHAR2**	1.93E+07	0.004%	1.91E+07	0.004%	4.25E+07	0.008%	7.02E+07	0.015%	**DHAR2**	0.008%
At1g07890	**APX1**	1.50E+09	0.305%	1.38E+09	0.322%	1.84E+09	0.351%	1.59E+09	0.339%	**APX1**	0.329%
At3g09640	**APX2**	-	-	-	-	-	-	-	-	**APX2**	0.000%
		identified proteins	5707	identified proteins	5838	identified proteins	6007	identified proteins	5963		
		**Sum (iBAQs)**	**4.91E+11**	**Sum (iBAQs)**	**4.30E+11**	**Sum (iBAQs)**	**5.23E+11**	**Sum (iBAQs)**	**4.70E+11**		

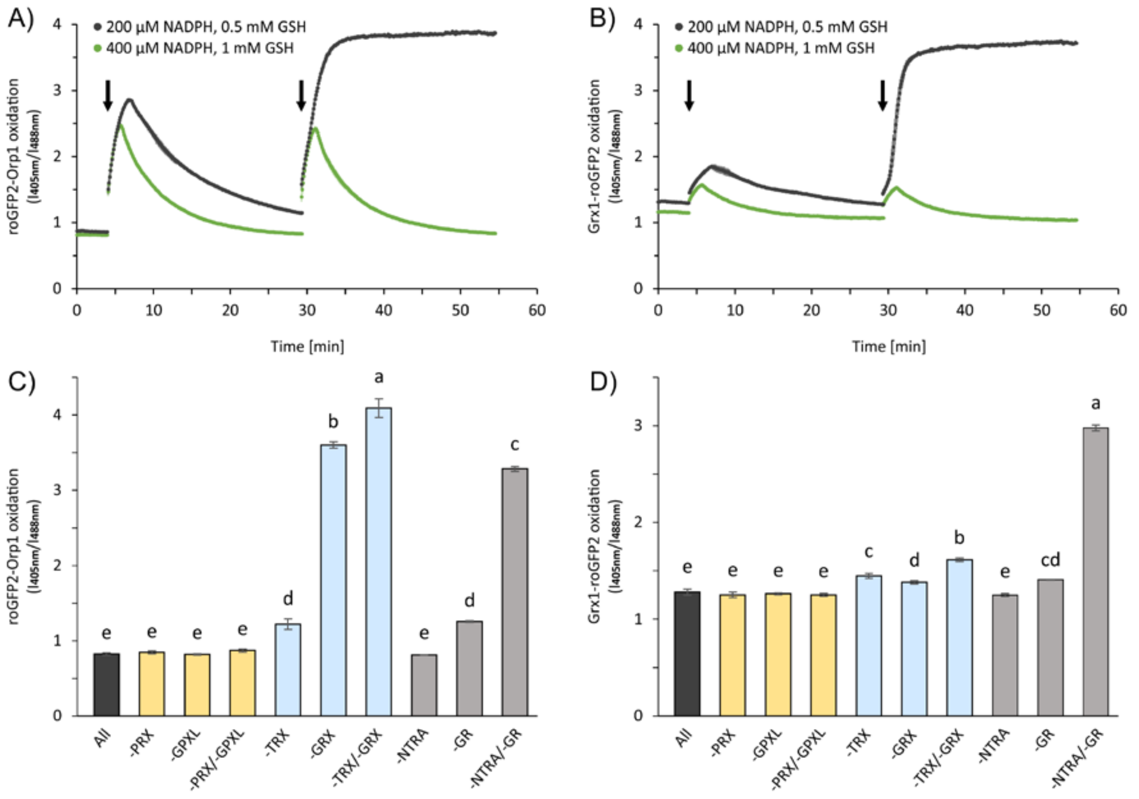

Figure S1: Changes in H_2O_2 concentration and E[GSH] in the reconstituted network including 500 µM GSH and 200 µM NADPH monitored by roGFP coupled to Orp1 or GRX1. Oxidation state was measured by laser scanning microscopy as ratio of the fluorescence emissions at the excitation wavelengths 405 nm and 488 nm. (A, B) The first 4 min show baseline recordings, then 100 µM H_2O_2 was added at two time points as indicated by black arrows. The green dataset was obtained in presence of doubled amount of NADPH and GSH in comparison to the black dataset. Response of roGFP2-Orp1 (A) and Grx1-roGFP2 (B) to H_2O_2-addition is shown. Mean ± SD, n=2. (C, D) Oxidation state of the sensors roGFP2-Orp1 (C) and Grx1-roGFP2 (D) after one hour equilibration of the reconstitution components observed during the initial 4 min baseline recordings before H_2O_2 was added. The redox state varied depending on the composition of the network. Yellow bars indicate the absence of thiol peroxidases, blue bars absence of transmitters and grey bars absence of input elements. Mean ± SD, n=4-20. ANOVA and Tukey post hoc test identified significant differences between the bars as indicated by letters ($p<0.05$).

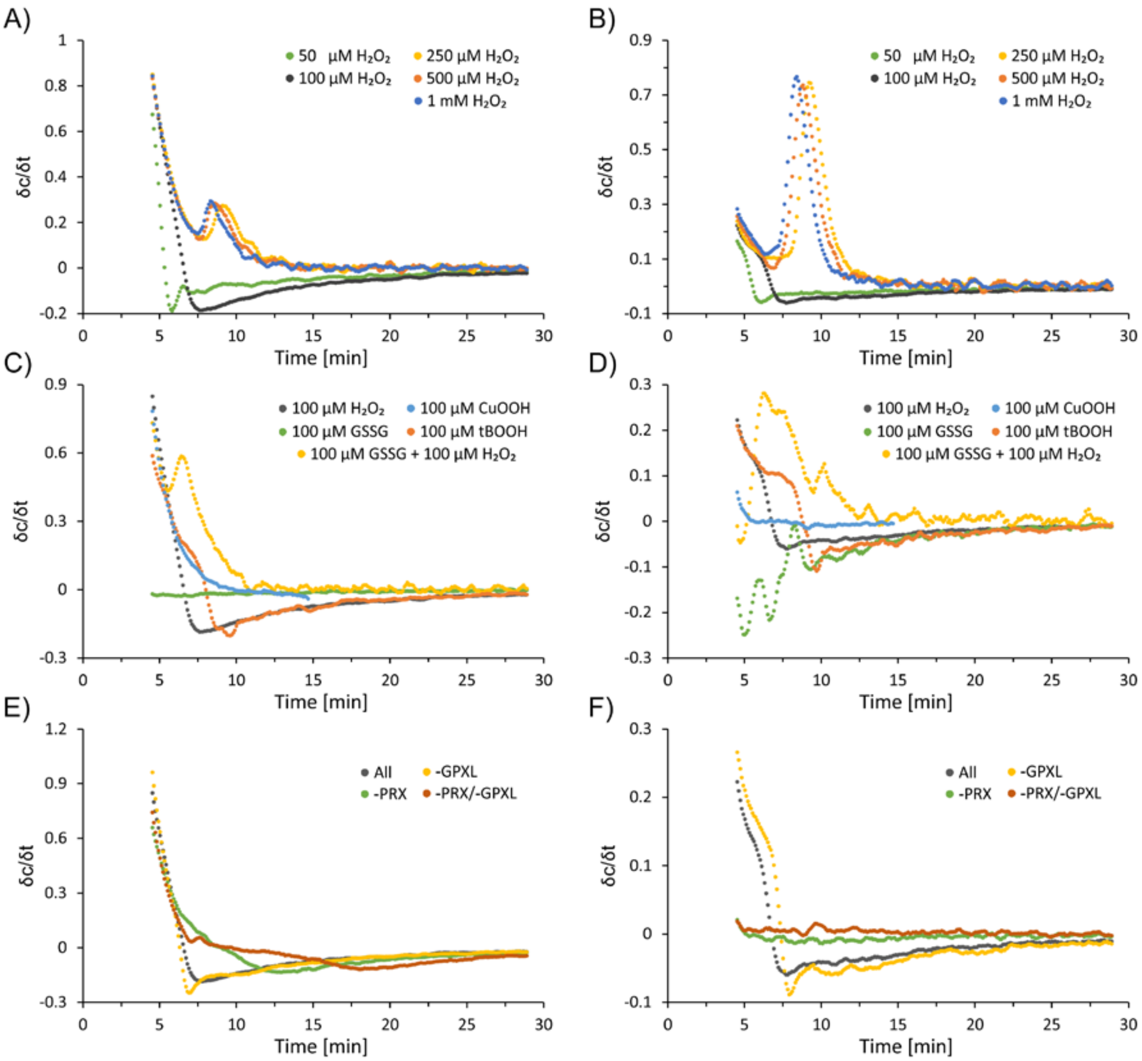

Figure S2: The graphs are derived from data shown in figure 2, which displays changes in H_2O_2 concentration and E[GSH] in the reconstituted network monitored by roGFP2 coupled to Orp1 or GRX1. Given are the slopes of ten floating datapoints (time intervals of 100 ms) on y-axis against median of corresponding timepoints on x-axis. Only data starting with addition of H_2O_2 in reaction mixture is shown. A, B) Response of roGFP2-Orp1 and Grx1-roGFP2 to increasing H_2O_2-concentrations, respectively. C, D) Comparison of the roGFP2-Orp1 and Grx1-roGFP2 oxidation state after addition of the different oxidants H_2O_2, GSSG, GSSG and H_2O_2, tBOOH and CuOOH, respectively. E, F) Impact of the presence or absence of thiol peroxidases in the reconstitution network on the oxidation state of the sensors roGFP2-Orp1 and Grx1-roGFP2, respectively. The redox state was monitored in the absence of either GPXL or PRX or GPXL/PRX.

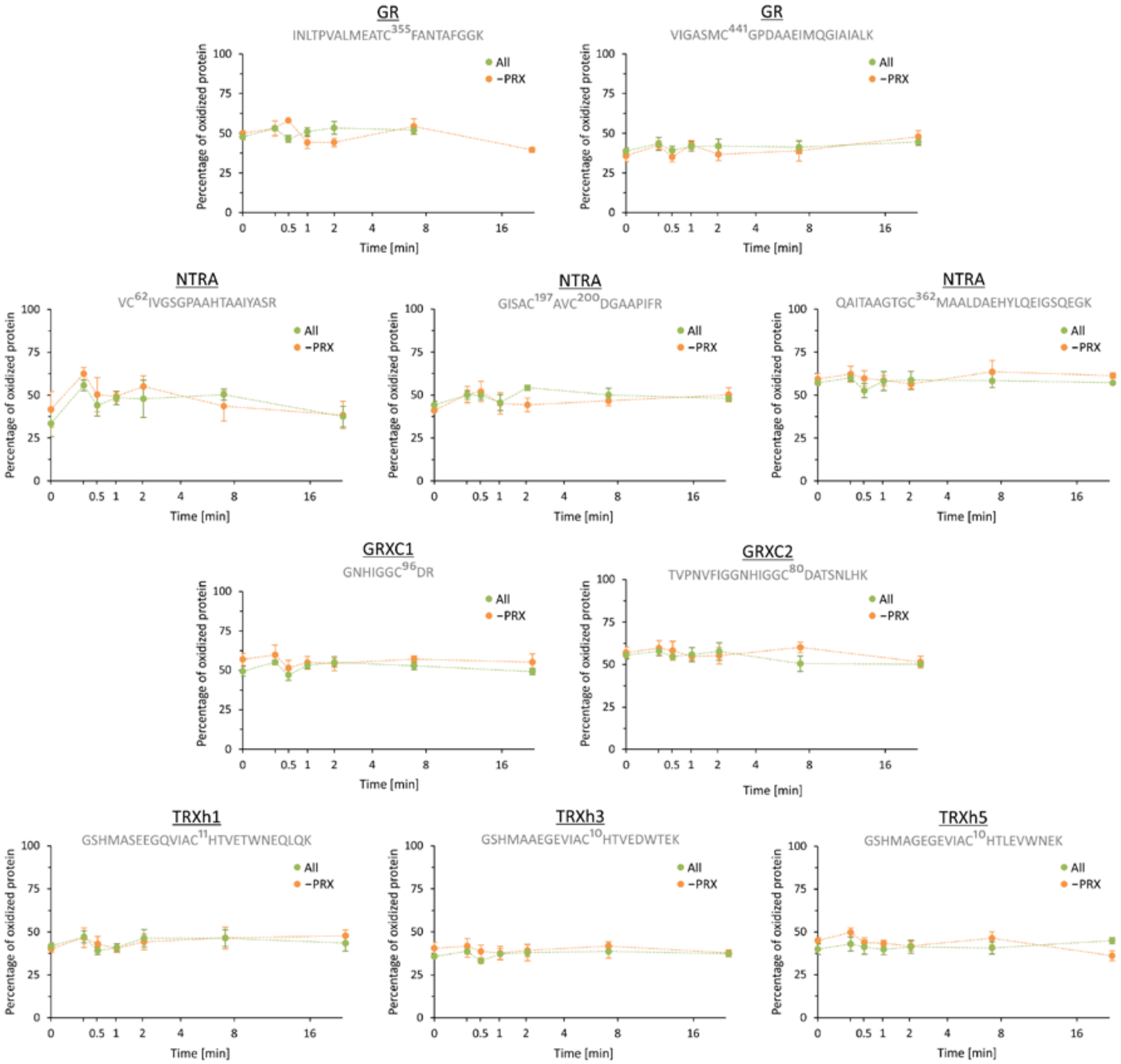

Figure S3: Time-dependent percentage of oxidized proteins in the reconstituted network in presence and absence of PRX after the addition of 100 µM H_2O_2. The oxidation states of GR, NTRA, TRXh1, TRXh3, TRXh5, GRXC1 and GRXC2 were determined by mass spectrometry at defined time points (0, 0.25, 0.5, 1, 2, 7 and 20 min) after adding H_2O_2 to the complete reconstitution system (green) or in absence of PRX (orange). The corresponding peptides are provided in grey letters, superscript numbers denominate the position of different cysteines. Mean ± SE, n=4. There were no significant statistical differences between samples with and without PRX (*t*-test, $p<0.05$) or between the different time points (ANOVA and Tukey post hoc test, $p<0.05$).

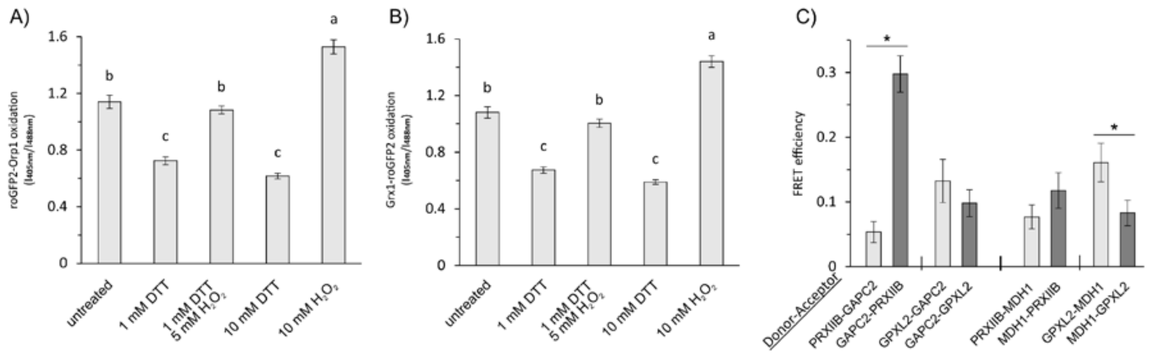

Figure S4: Cytosolic redox states and FRET-measurements in protoplasts. roGFP2-Orp1 (A) and Grx1-roGFP2 (B) were expressed transiently in *A. thaliana* protoplasts under control of the ubiquitin 10 promoter and the redox state of the sensors was measured under reducing and oxidizing conditions as indicated. Pre-reduction by 1 mM DTT for 30 min was followed by oxidation with 5 mM H_2O_2 for another 30 min. Each sample was incubated for 30 min before starting the measurements. Mean ± SE, n=72-106. Significant differences were identified by ANOVA and Tukey-test and indicated by different letters ($p<0.05$). (C) *In vivo* FRET-measurements between the targets MDH1, GAPC2 and the thiol peroxidases in protoplasts. mTurquoise2 as donor or YFP as acceptor were fused to targets and the thiol peroxidases in both directions, so that acceptor and donor were switched to address differences in the expression of donor and acceptor and their impact on the FRET-efficiency due to a possible excess of donors (grey bars: Thiol peroxidase as donor; dark grey bars: Targets as donor), and transiently expressed under control of the CaMV35S-promoter. Mean ± SE, n=32-42. *t*-test was performed to identify significant differences ($p<0.05$).

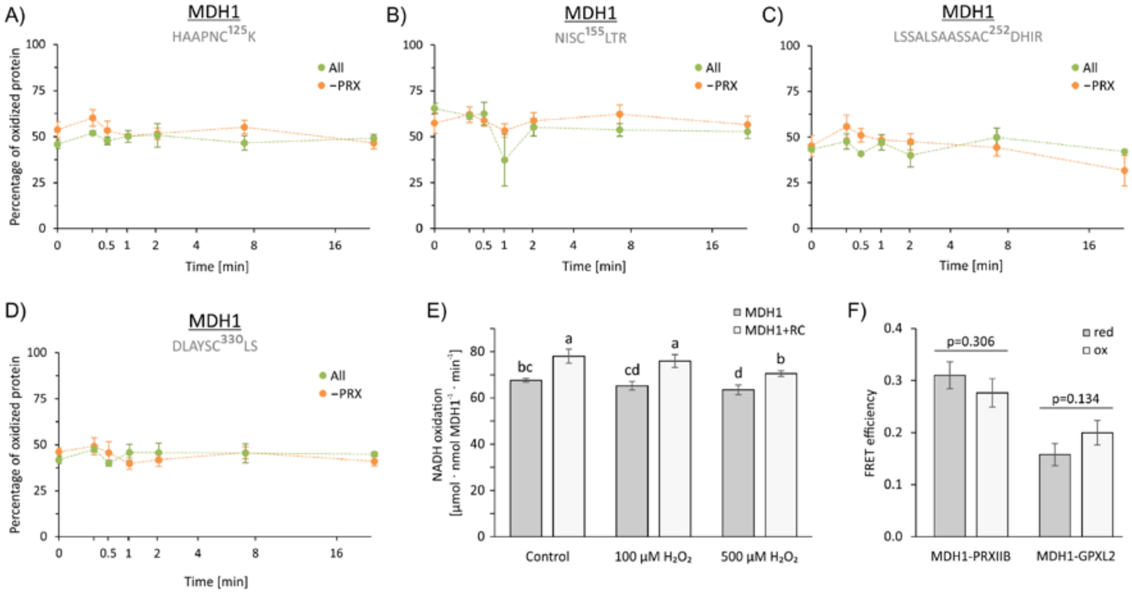

Figure S5: MDH1 as target of thiol peroxidases. (A-D) Time-dependent percentage of oxidized MDH1 in the reconstituted network in presence (green) and absence (orange) of PRX after the addition of 100 µM H_2O_2. The identified peptide is given in grey letters, superscript numbers denominate the position of different cysteines. The oxidation state was determined via mass spectrometry at defined time points (0, 0.25, 0.5, 1, 2, 7 and 20 min) after H_2O_2-addition. Mean ± SE, n=4. There were no significant statistical differences between samples with and without PRX (*t*-test, $p<0.05$) or between the different time points (ANOVA and Tukey post hoc test, $p<0.05$). (E) The MDH1-activity was measured as change in absorbance at 340 nm corresponding to the NADH-concentration. Measurements were performed in absence (grey bars) and presence (white bars) of the reconstitution components (RC) and as a control, activity was also measured for the protein mix of the reconstituted system lacking MDH1, which showed no activity. All samples contained 500 µM GSH and 200 µM NADPH. The inhibitory effect of 100 µM H_2O_2 and 500 µM H_2O_2 was analyzed after 30 min incubation. MW±SD, n=6. Statistical significance was analyzed by ANOVA and Tukey post hoc test ($p<0.05$). (F) *In vivo* FRET-measurements between MDH1 and the thiol peroxidases in protoplasts. mTurquoise2 as donor or eYFP as acceptor were fused to MDH1 and the thiol peroxidases, respectively, and transiently expressed under control of the CaMV35S-promoter. The cells were reduced by 1 mM DTT, the FRET-efficiency was determined by sensitized acceptor emission. Then the reduced cells were oxidized by 5 mM H_2O_2 and the FRET-efficiency was determined for the oxidized cells. Mean ± SE, n=23-37. *t*-test was performed to identify significant differences.

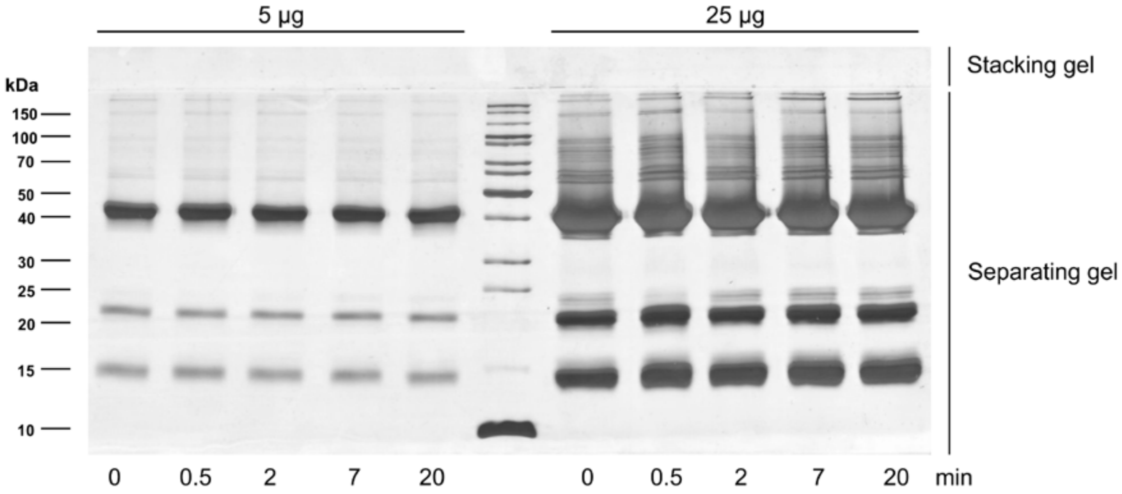

Figure S6: Redox-dependent protein conformation and aggregate formation in reconstituted system after oxidation with H_2O_2. The protein mixtures with 500 µM GSH, 200 µM NADPH and 140 µM NAD were subjected to 100 µM H_2O_2. After 0, 0.25, 0.5, 2, 7 and 20 min, samples were alkylated. 5 or 25 µg total protein were separated in a non-reducing SDS-PAGE and visualized by silver staining.
